# Pyridyl-Thiourea Ruthenium and Osmium Complexes: Coordination of Ligand and Application as FLP Hydrogenation Catalysts

**DOI:** 10.3390/molecules30163398

**Published:** 2025-08-16

**Authors:** Alejandro Grasa, Roisin D. Leavey, Fernando Viguri, Ricardo Rodríguez, Pilar Lamata

**Affiliations:** Instituto de Síntesis Química y Catálisis Homogénea (ISQCH), CSIC—Universidad de Zaragoza, Departamento de Química Inorgánica, Pedro Cerbuna 12, 50009 Zaragoza, Spain; agrasa@unizar.es (A.G.); roisinhazell@gmail.com (R.D.L.)

**Keywords:** thiourea, ruthenium, osmium, frustrated Lewis pairs, H-H activation

## Abstract

Pyridyl-thiourea complexes of formula [(Cym)MCl(κ^2^*N*_py_,*S*-**H_2_NNS**)][SbF_6_] (Cym = *η*^6^-*p*-MeC_6_H_4_*i*Pr; **H_2_NNS** = *N*-(*p*-tolyl)-*N′*-(2-pyridylmethyl)thiourea); M = Ru (**1**), Os (**2**)) were synthesized by reacting the corresponding metal dimers [{(Cym)MCl}_2_(*μ*-Cl)_2_] with **H_2_NNS** in the presence of NaSbF_6_. Subsequent chloride abstraction with AgSbF_6_, followed by NH deprotonation using NaHCO_3_, afforded the cationic complexes [(Cym)M(κ^3^*N*_py_,*N*_amide_,*S***-HNNS**)][SbF_6_] (M = Ru (**5a**), (**5c**); M = Os (**6a, 6c**)) and [(Cym)M(κ^2^*N*_amide_,*S***-HNNS**)][SbF_6_] (M = Ru (**5b**); M = Os (**6b**)). The proposed structures for the prepared compounds are based on NMR data. Complexes **5a**, **5b,** and **6a**, **6b** evolve to the thermodynamically more stable species **5c** and **6c**, respectively, in which the deprotonated ligand **HNNS** adopts a κ^3^*N*_py_,*N*_amide_,*S* coordination mode. Complexes **5c** and **6c** activate H_2_, behaving as frustrated Lewis pair (FLP) species, and catalyze (**5c** and/or **6c**) the hydrogenation of polar multiple bonds, including the C=N bonds of *N*-benzylideneaniline and quinoline, the C=C bond of methyl acrylate, and the C=O bond of 2,2,2-trifluoroacetophenone.

## 1. Introduction

There are known examples of intra- and intermolecular combinations of Lewis acids and bases that, in solution, for steric, electronic reasons, or both, do not form the corresponding Lewis adducts. These acid-base pairs are referred to as frustrated Lewis pairs (FLP). A milestone in the development of such systems was the discovery in 2006 by Stephan and coworkers that such species, in which no metal was present, were capable of activating the hydrogen molecule heterolytically and reversibly under mild conditions [1]. A few years later, it was found that the acidic and basic components of FLPs could activate many other small (CO_2_, CO, SO_2_, N_2_O, NO) and organic (olefins, alkynes) molecules in a cooperative and concerted manner, following new reaction pathways [2,3,4,5,6,7,8,9,10,11,12].

Much less developed, but becoming increasingly important, are FLPs in which one of the two FLP components is a transition metal fragment. Pioneering work from Wass [13] and Erker’s [14] groups on zirconium-phosphane combinations was shortly followed by notable metal examples, demonstrating the potential of transition-metal frustrated Lewis pairs (TMFLPs) in small molecule activation and catalysis. Introducing a transition metal into the system provides the FLP with greater structural diversity, enabling access to transition metal fundamental catalytic reactions [15,16,17,18,19,20,21,22,23,24,25,26,27,28,29,30,31].

**Figure 1:**
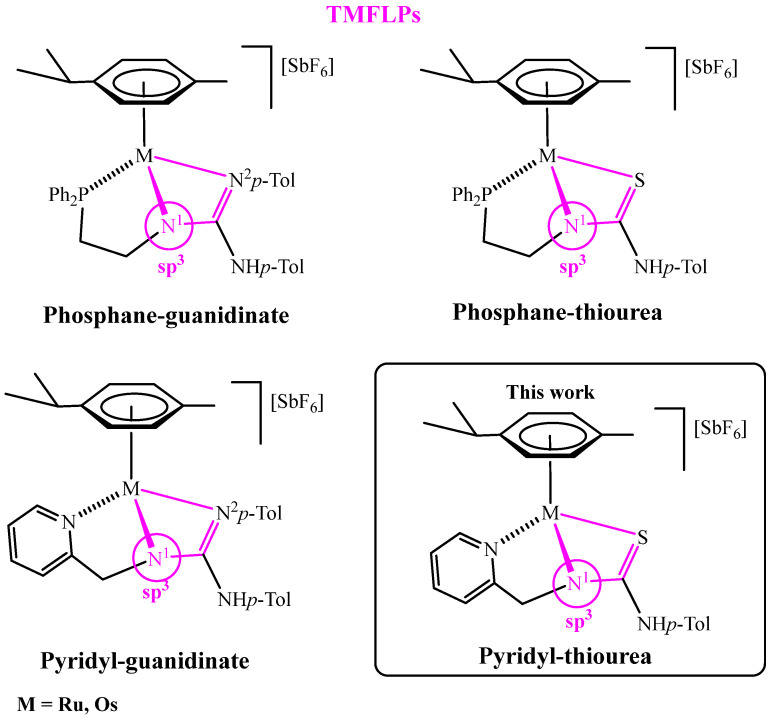
TMFLPs compounds based on phosphane-guanidinate, pyridyl-guanidinate, and phosphane-thiourea ligands.

In particular, we have recently prepared the phosphane-guanidinate, pyridyl-guanidinate, and phosphane-thiourea complexes of ruthenium and osmium [32,33,34] in which the ligands adopt a *fac* coordination (Figure 1). This coordination forces the central nitrogen atom (N^1^) to take on sp^3^ hybridization. Under these conditions, the four-membered cycles M−N^1^−C−N^2^ and M−N^1^−C−S support a strong ring strain that can be relaxed upon breaking the M−N^1^ (thiourea complexes) or M−N^2^ bond (guanidinate complexes), giving rise to active FLP species. However, in this context, in the course of the study of the catalytic activity in hydrogenation reactions, we detected that in the pyridyl-guanidinate complexes, the presence of protons in the 2 and 6 positions of the *p*-tolyl substituent gives rise to metallation reactions that preclude FLP reactivity, due to the change in the metalacycle from four members into a five-membered M−N^1^−C−N^2^−C [32].

**Figure 2:**
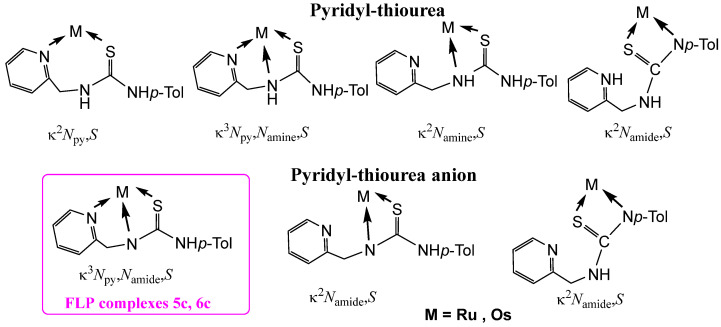
Coordination modes of pyridyl-thiourea ligands in the work.

Taking these aspects into consideration, in this work we report the synthesis and characterization of pyridyl-based complexes in which the guanidine moiety (N^2^*p*-Tol) of the previously studied tridentate ligand has been replaced by a more robust thiourea fragment (Figure 1) [35,36,37,38]. A characteristic feature of these compounds—whether bearing the neutral **H_2_NNS** or the anionic **HNNS** pyridyl-thiourea ligand—is the existence of a dynamic equilibrium between molecular species exhibiting distinct coordination modes (Figure 2). When the deprotonated monoanionic ligand **HNNS** adopts a κ^3^*N*_py_,*N*_amide_,*S* coordination mode (as in complexes **5c** and **6c**), the resulting M−N_amide_−C−S four-membered ring can undergo structural relaxation via cleavage of the metal–amide nitrogen bond. This process leads to the generation of frustrated Lewis pair (FLP) species. The FLP behaviour of complexes **5c** and **6c** has been investigated in the context of hydrogenation catalysis, demonstrating their ability to heterolytically activate molecular hydrogen and promote the reduction in unsaturated substrates.

## 2. Results and Discussion

### 2.1. Synthesis of the Ligand

**Scheme 1:**
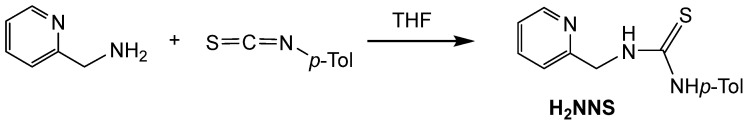
Preparation of **H_2_NNS** ligand.

The pyridyl-thiourea ligand **H_2_NNS** has been prepared in high yield by reacting 2-pyridylmethanamine with *p*-tolyl-isothiocyanate in dry THF (Scheme 1) following literature procedures (see Materials and Methods) [32,33,34,39].

### 2.2. Synthesis of the Chlorido Complexes [(Cym)MCl(κ^2^N_py_,S-H_2_NNS)][SbF_6_] (M = Ru (***1***), Os (***2***))

**Scheme 2:**
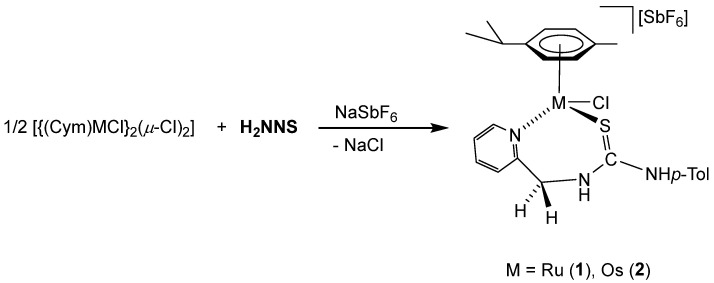
Preparation of complexes **1** and **2**.

The chlorido complexes **1** and **2** were prepared, with a yield of 96% (**1**) and 90% (**2**), by treating the dimers [{(Cym)MCl}_2_(*μ-*Cl)_2_] [40,41] with stoichiometric amounts of the ligand **H_2_NNS** in methanol in the presence of NaSbF_6_ (Scheme 2).

The complexes were characterised by analytical and spectroscopic means (see Materials and Methods). Two-dimensional homonuclear and heteronuclear correlations verified the assignment of the NMR signals. At this point, it should be noted that for compound **1**, the presence in the ^1^H NMR spectra of only one species has been observed in CD_2_Cl_2_, (CD_3_)_2_CO, and CD_3_OD. However, for the compound **2** in CD_2_Cl_2_, signals corresponding to four species are identified, and these evolve to a unique set of signals in more polar solvents, such as (CD_3_)_2_CO and CD_3_OD. This behaviour is consistent with a change in the coordination mode of the **H_2_NNS** ligand. The mass spectrum of ruthenium compound **1** agrees with the structure shown in Scheme 2, where the chlorine atom is coordinated to the ruthenium centre. The similarity of the NMR spectra of compounds **1** and **2** in polar solvents allows us to propose the same structure for the two compounds (Scheme 2). Coordination of the pyridyl nitrogen to the metal in complexes is supported by a strong deshielding of the H_6_ proton of the pyridyl moiety, from 8.44 (free ligand) to 9.52 (complex **1**) and 9.42 ppm (complex **2**). Additionally, in a similar way to the related ruthenium and osmium phosphane-thiourea complexes published by us [32], the coordination through the sulphur atom in both complexes gives rise to the seven-membered metallacycles. The metal is a stereogenic centre, and accordingly, the methylene hydrogens are diastereotopic and resonate as a pair of signals in each case, 5.69 and 4.79 ppm (complex **1**) and 5.65 and 4.74 ppm (complex **2**) (see Materials and Methods).

### 2.3. Synthesis of Dicationic Ruthenium Complexes ***3***

**Scheme 3:**
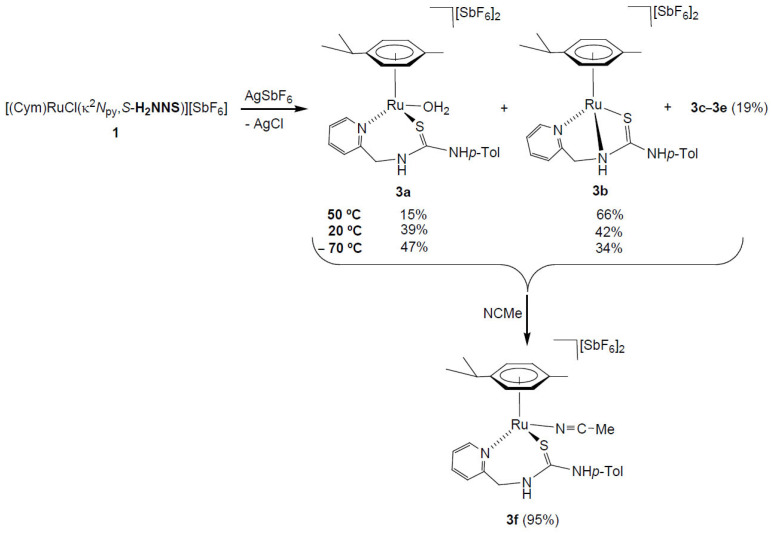
Preparation of dicationic ruthenium complexes **3**.

A mixture of dicationic complexes **3a**–**3e** was obtained by treating the chlorido complex **1** with AgSbF_6_, in acetone. The ^1^H NMR spectrum of the isolated solid in (CD_3_)_2_CO shows the presence of two major compounds, **3a** (39%) and **3b** (42%), together with three minor compounds, **3c**–**3e** (19%) (Scheme 3, Materials and Methods and Supplementary Materials). The presence of trace amounts of water in the solvent is sufficient for **3a** aqua-complex to form. By variable temperature ^1^H NMR in (CD_3_)_2_CO we studied the behaviour in solution. At −70 °C, the spectrum showed the presence of the five compounds **3a** (47%), **3b** (34%), and **3c**–**3e** (19%). When the temperature was raised to 50 °C, the proportion of compound **3a** decreased at the expense of **3b** (**3a** (15%), **3b** (66%), and **3c**–**3e** (19%)) (Scheme 3). In addition, when 100 μL of NCMe are added to the mixture of five compounds in (CD_3_)_2_CO, the formation of a new compound with NCMe coordinated of stoichiometry [(Cym)Ru(NCMe)(κ^2^*N*_py_,*S*-**H_2_NNS**)][SbF_6_]_2_ (**3f**, 95% abundance) is essentially observed (Scheme 3). This behaviour indicates the presence of different dicationic species (**3a**–**3e**) according to different forms of coordination κ^2^ or κ^3^ of the **H_2_NNS** ligand and the coordination or not of the solvent molecules, such as H_2_O [32,33].

For the major compounds **3a** and **3b** we propose the structures drawn in Scheme 3, based on related ruthenium complexes with pyridyl-guanidine [33] [(Cym)Ru(H_2_O)(κ^2^*N*,*N*-**H_2_NNN**)][SbF_6_]_2_ and phosphane-thiourea [32] [(Cym)Ru(κ^3^*P*,*N,S*-**H_2_PNS**)][SbF_6_]_2_ ligands, informed previously by us. A significant difference between these two compounds is the chemical shift of the methylene carbon CH_2_, 44.25 ppm in **3a** and 61.40 ppm in **3b.** The chemical shifts of the methylene carbons of complexes prepared in this work resonate in the 61.20–63.08 ppm range when the ligand is κ^3^ coordinated and at a lower chemical shift (44.25–53.24 ppm) when it is κ^2^ coordinated. Most probably, in complex **3b**, where the sp^3^ nitrogen is coordinated to the metal, it shares a lower electron density with the methylene carbon.

### 2.4. Synthesis of the Dicationic Complexes [(Cym)M(NCMe)(κ^2^N_py_,S-H_2_NNS)][SbF_6_]_2_ (M = Ru (***3f***), Os (***4a***))

**Scheme 4:**
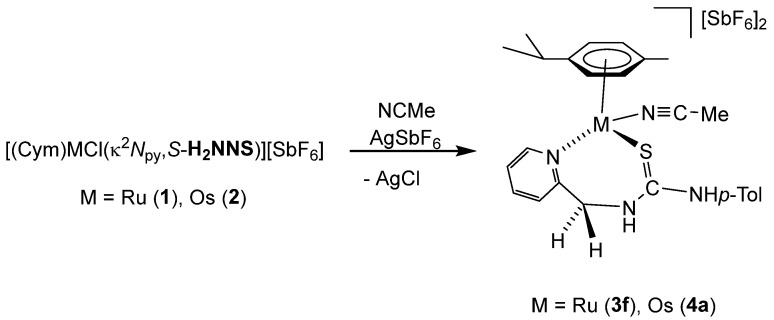
Synthesis of complexes **3f** and **4a**.

The chloride ligand in complexes **1** and **2** was eliminated as AgCl by treatment with AgSbF_6_ in NCMe. The presence of NCMe allows the isolation of the dicationic complexes [(Cym)M(NCMe)(κ^2^*N*_py_,*S*-**H_2_NNS**)][SbF_6_]_2_ (M = Ru (**3f**), Os (**4a**)) in high yield (Scheme 4).

Complexes **3f** and **4a** were characterised by analytical and spectroscopic means (see Materials and Methods). In proton NMR, strong deshielding was observed for the H_6_ proton of the pyridine moiety, *δ*H_6_ = 9.17 ppm (**3f**) and 9.12 ppm (**4a**). The NH bonded to the *p*-tolyl group resonates at 8.22 ppm (**3f**) and 8.16 ppm (**4a**), and the NH bonded to the methylene group resonates as a broad singlet at 7.13 ppm (**3f**) and as a broad triplet at 7.24 ppm (**4a**), due to the coupling with the CH_2_ protons. The observed NOEs are in agreement with the proposed structure, where the ligand is κ^2^ coordinated through the pyridyl N and S atoms.

### 2.5. Synthesis of the Monocationic Ruthenium Complexes ***5***

**Scheme 5:**
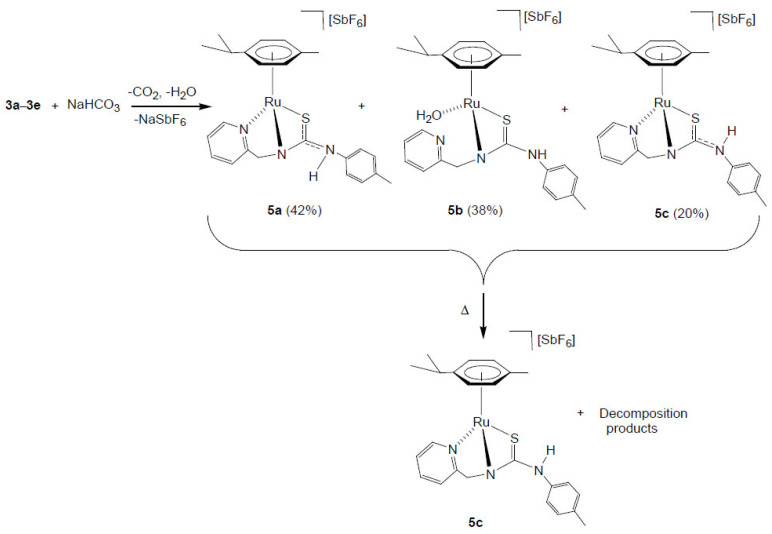
Synthesis of the monocationic ruthenium complexes **5**.

The addition of solid NaHCO_3_ to a solution of a mixture of complexes **3a**–**3e** in methanol (Scheme 5) results in the formation of the monocationic complexes **5a**–**5c** in which the coordinated ligand **H_2_NNS** is monodeprotonated.

The initial proportion of complexes **5a**/**5b**/**5c** (42/38/20 molar ratio) changes to 27/24/49, with a 13% decomposition of the products, after maintaining the acetone solution for 5 h at 50 °C. Reacting the mixture of complexes **5a**–**5c** with H_2_ at RT, in THF-*d*_8_/D_2_O, gives the compound **5c** pure (see below).

**Figure 3:**
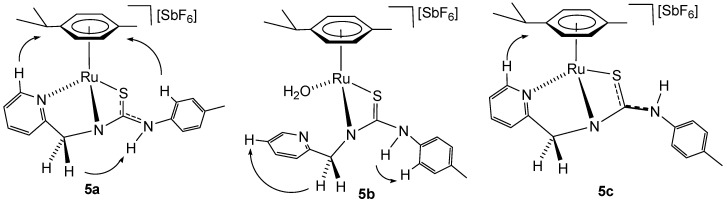
Selected NOE for the complexes **5a**–**5c**.

Complexes **5a**–**5c** were characterised by analytical and spectroscopic means (see Materials and Methods and Supplementary Materials). Coordination of ligand by the pyridyl nitrogen in complexes **5a** and **5c** is supported by the deshielding of the H_6_ proton of the pyridyl moiety to 9.28 (complex **5a**) and 9.05 ppm (complex **5c**) and can be assessed through NOE experiments (Figure 3). In complex **5b**, the chemical shift of H_6_ from the pyridyl group, together with the no NOE observed between this proton and the *p*-cymene group, indicates that the pyridyl moiety is not coordinated to ruthenium. In addition, NOE was observed between the CH_2_ protons and the H_5_ of the pyridyl moiety (Figure 3). The absence of chemical coupling between CH_2_ and NH protons indicates that NH is bonded to the *p*-tolyl group in the three complexes **5a**–**5c**.

All this data support the proposed structures for compounds **5a** and **5c**, in which the pyridyl-thiourea **HNNS** ligand is coordinated in a κ^3^ fashion, whereas in compound **5b**, a κ^2^ coordination mode involving the amide nitrogen and sulphur atoms is observed. A water molecule likely completes the coordination sphere of the metal centre in **5b**. This interpretation is consistent with the chemical shifts observed in the ^13^C NMR spectra for the methylene carbon; compounds **5a** and **5c** display similar resonances at 62.21 ppm and 61.20 ppm, respectively, while compound **5b** shows a signal significantly upfield at 48.38 ppm.

We propose that compounds **5a** and **5c** exist as two distinct rotamers around the C(S)–N(H) bond, separated by a relatively high rotational energy barrier. This is consistent with the sharp signals observed in the NMR spectra for **5a** and **5c**, indicative of slow exchange on the NMR timescale. Additional support for this structural assignment comes from NOE experiments. Only in compound **5a**, an NOE between the aromatic proton of the *p*-tolyl group and the *p*-cymene ligand is observed (Figure 3). These findings agree with previously reported DFT calculations on structurally related ruthenium complexes [32,33]. For example, in the case of [(Cym)Ru(κ^3^*P*,*N*,*S*-**HPNS**)][SbF_6_], containing a phosphane-thiourea ligand [32], a fluxional behaviour is observed in solution. The C(S)–N(H) bond in this complex exhibits an intermediate bond length between a typical single and double bond, and two conformers are detected in the NMR spectra. Similarly, in one of the crystallographically characterised conformers of [(Cym)RuCl(κ^2^*N*,*N*-**H_2_NNN**)][SbF_6_] with a pyridyl-guanidine ligand [33]. the C–N bond also displays a partial double-bond character, further reinforcing the hypothesis of restricted rotation and conformational stability in these types of complexes.

### 2.6. Syntheses of the Monocationic Osmium Complexes ***6***

**Scheme 6:**
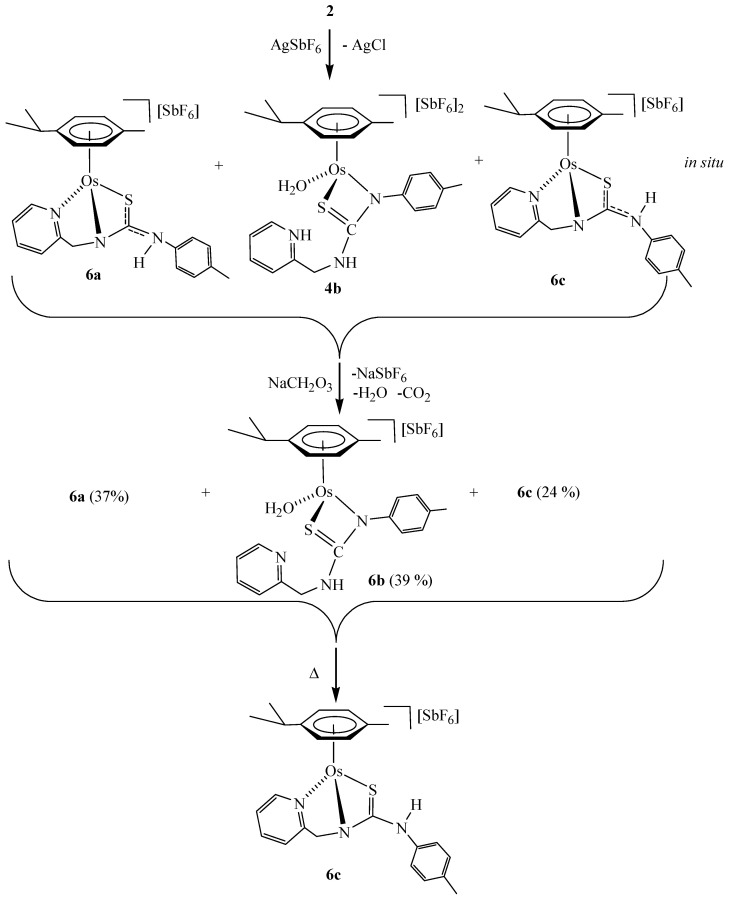
Synthesis of the monocationic osmium complexes **6**.

Osmium compound [(Cym)OsCl(κ^2^*N*_py_,*S***-H_2_NNS**)][SbF_6_] (**2**) reacts with AgSbF_6_ in acetone, rendering a mixture of the monocationic complexes [(Cym)Os(κ^3^*N*_py_*,N*_amide_,*S***-HNNS**)][SbF_6_] (**6a** and **6c**) and the dicationic complex [(Cym)Os(H_2_O)(κ^2^*N*_amide_,*S***-H_2_NNS**)][SbF_6_]_2_ (**4b**). Addition of NaHCO_3_ to the mixture yields the monocationic complexes **6a**-**6c.** The experimental data support the structural formulations (Scheme 6).

The initial proportion of complexes **6a**/**6b**/**6c** (37/39/24 molar ratio) changes to 12/16/72, after maintaining the methanol solution at 60 °C for 24 h. The lower solubility of compound **6c** in methanol allowed isolating it as a yellow solid with 98% purity (see Materials and Methods).

**Figure 4:**
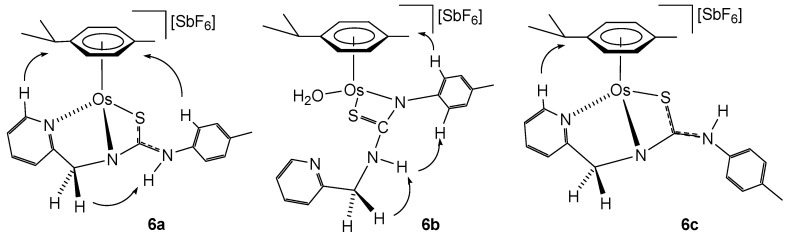
Selected NOE for the complexes **6a**–**6c**.

Osmium complexes **6a**–**6c** were characterised by analytical and spectroscopic means. Spectroscopic data indicate that compounds **6a** and **6c** show a similar structure to ruthenium compounds **5a** and **5c,** respectively. Coordination of the pyridyl nitrogen of the ligand at the metal in complexes **6a** and **6c** is supported by a deshielding of the H_6_ proton of the pyridyl moiety to 9.00 (complex **6a**) and 8.87 ppm (complex **6c**). In complex **6b**, the chemical shift of H_6_ from the pyridyl group (8.39 ppm) indicates that the pyridyl moiety is not coordinated to osmium, and the NH group resonates as a broad triplet at 4.73 ppm, indicating that the NH is bonded to the methylene group. The chemical shift of the methylene carbon ^13^C NMR in compounds **6a**, **6b**, and **6c** is 62.46, 46.09, and 62.66 ppm, respectively. The most representative observed NOEs are shown in Figure 4, which are in agreement with the proposed structures.

At this point, it should be noted that the ^1^H NMR spectrum of osmium compound **4b** shows the same signals as **6b** except for the NH and H_6_ of the pyridinium group in **4b**. For compound **4b**, a broad ^1^H singlet at 12.45 ppm coupled with H_6_ is attributed to the NH functionality of the pyridinium group. Consequently, the H_6_ of the pyridinium group resonates at 8.35 ppm as a pseudo triplet (*J* = 5.0 Hz).

**Scheme 7:**
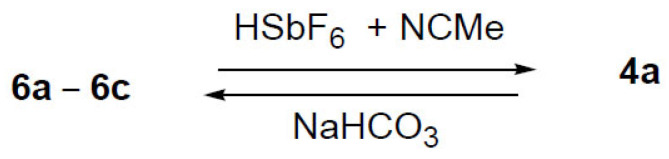
Conversion reactions between **4a** and **6a**–**6c**.

Additionally, the reaction for the formation of compound **4a** from **6a**–**6c** in the presence of HSbF_6_ and NCMe was carried out, as well as the formation of **6a**–**6c** from **4a** in methanol in the presence of NaHCO_3_ (Scheme 7).

**Figure 5:**
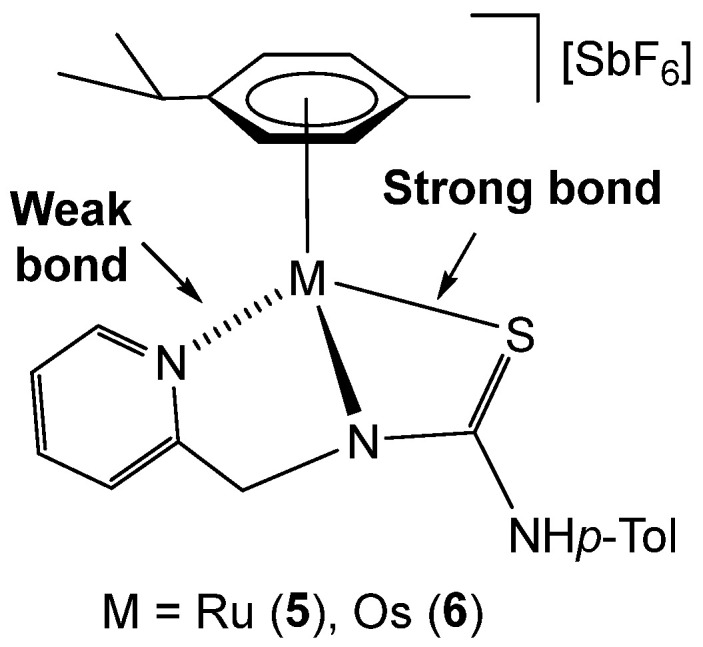
Relative bond strength in complexes **5** and **6**.

Concerning the observed equilibria and structural diversity in complexes **5** and **6**, the greater lability of the pyridyl–metal bond in the pyridyl-thiourea complexes, compared to the related pyridyl-guanidinate analogues previously reported [32,33,34], may be attributed to the stronger M–S bond in the thiourea moiety, which can reinforce coordination to the metal centre and disfavour simultaneous tight binding of all three donor atoms (Figure 5). This difference in bonding behaviour appears to facilitate reversible coordination-decoordination processes of the pyridyl group, which are not observed in the more rigid pyridyl-guanidinate systems. This hemilability plays a critical role in the formation of frustrated Lewis pair species. At this point, it is necessary to point out that some FLP systems can show their activity even if the classical Lewis acid–base adduct is stable, provided that the dissociated form is thermally accessible. Such a type of FLP is denominated as a masked FLP [26].

### 2.7. Catalytic Hydrogenation Assays

The chemical behaviour of complexes **5** and **6**, previously discussed, allows us to have complexes **5c** and **6c** that show structural characteristics of FLP. This is a consequence of the strained four-membered M−N−C−S metallacycle in the *fac* κ^3^*N*,*N*,*S* coordination mode of the ligand. This metallacycle can be opened, giving rise to FLP species, in which the metal and nitrogen atoms play the roles of Lewis acid and Lewis base components, respectively, activating the hydrogen molecule. Complexes **5** and **6** have been tested as catalysts in the hydrogenation of benchmark substrates such as the C=C double bond in the methyl acrylate [42,43], the C=O bond in the 2,2,2-trifluoroacetophenone [44,45,46,47,48] and the C=N bond in the *N*-benzylideneaniline and quinoline [44,46,49]. It should be noted that similar results are obtained using mixtures of complexes **5** and **6**, or pure complexes **5c** and **6c**. The hydrogenation reactions were carried out at 60 °C, in THF-*d*_8_ with a catalyst/substrate molar ratio of 1/20 under 5 bar of H_2_. For ruthenium complexes **5**, from 2.5 to 9 days were needed to achieve complete conversion in the hydrogenation of methyl acrylate, *N*-benzylideneaniline, and quinoline (Table 1, entries 1–3). For the substrate 2,2,2-trifluoroacetophenone, no quantitative conversions are achieved, and a decrease in the rate of the reaction is observed while free *p*-cymene is progressively formed due to catalyst decomposition (Table 1, entry 4). The osmium complex **6c** showed lower activity than complex **5c** (Table 1, entry 6), and in a similar way to the ruthenium catalyst, no quantitative conversions are obtained with substrates with carbonyl groups (Table 1, entries 5 and 7). Only complexes **5** or **6** were detected under catalytic conditions, indicating that catalyst hydrogenation is the slow step in the catalytic cycle, being the resting state **5c** or **6c**.

**Scheme 8:**
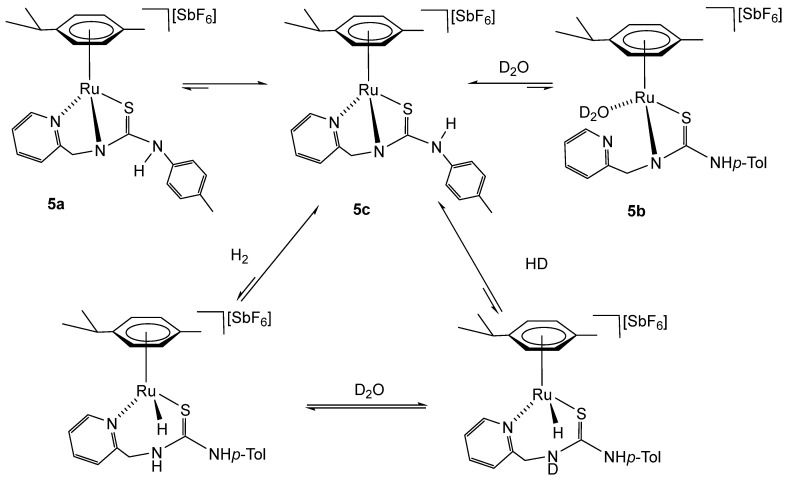
Reaction with H_2_ of complexes **5a**–**5c** in THF-*d*_8_/D_2_O.

When we explored the reactivity of complexes **5** and **6c** with molecular hydrogen (5 bar, 60 °C) in THF-*d*_8_, the corresponding hydride complex [(Cym)MH(κ^2^*N*_py_,*S*-**H_2_NNS**)][SbF_6_] was not observed. Then, we tested the isotopic reaction exchange from H_2_ to HD in the presence of D_2_O as a deuterium source. Effectively, at RT, this exchange reaction is observed when compounds **5** and **6c** are exposed to 5 bar of H_2_ in a mixture of THF-*d*_8_/D_2_O (0.35 mL/0.10 mL) for 30 min and 5 days, respectively. The appearance of HD implies that the activation of H_2_, leading to the formation of hydride complexes [(Cym)MH(κ^2^*N*_py_,*S*-**H_2_NNS**)][SbF_6_], is a reversible reaction, and the equilibrium is strongly shifted towards the starting complexes (Scheme 8). Furthermore, as we have previously mentioned, the most stable compound **5c** is obtained purely by reacting mixture **5a**–**5c** in THF-*d*_8_/D_2_O, 0.35 mL/0.1 mL, with H_2_ at RT for 5 days. Most probably, complex **5** reacts with H_2_ to give the hydride species, and this favours the formation of **5c** complex at the expense of **5a** and **5b** (Scheme 8).

The lability of the pyridyl–metal bond and the resulting formation of species such as **5b**, featuring a κ^2^ coordinated ligand, may account for the progressive decomposition of the catalyst over time and thus, for the relatively slow hydrogenation rates observed under catalytic conditions. Accordingly, species with carbonyl groups are well suited to compete with the pyridine group in the coordination to the metal and modify the catalyst (Table 1, entries 4, 5, and 7). The characterisation of compounds **5b** and **6b** provides further insight into the delicate equilibrium between different coordination modes and highlights that FLP behaviour in these systems arises from a finely tuned balance of bond strengths between the metal centre and the three donor atoms of the ligand in a *facial* κ^3^ arrangement. Slight deviations in this balance, particularly a weakening of the metal–pyridyl interaction, can shift the complex toward less stable κ^2^ species, which compromises both catalytic activity and long-term catalyst stability. These findings underscore the importance of ligand design in TMFLP systems, where controlled hemilability can promote cooperative reactivity, but excessive flexibility may lead to ligand decoordination and catalyst degradation.

## 3. Conclusions

A key feature of ruthenium and osmium complexes bearing pyridyl-thiourea ligands is the remarkable versatility in coordination modes exhibited by the **H_2_NNS** and **HNNS** ligands. These ligands can bind to the metal centre in several fashions, including κ^2^*N*_py_,*S* (complexes **1**, **2**, **3a**, **3f**, **4a**), κ^3^*N*_py_,*N*_amine_,*S* (complex **3b**), κ^3^*N*_py_,*N*_amide_,*S* (complexes **5a**, **5c**, **6a**, **6c**) and κ^2^*N*_amide_,*S* (complexes **4b**, **5b**, **6b**). Among these, complexes [(Cym)M(κ^3^*N*_py_*,N*_amide_*,S-***HNNS**)][SbF_6_] (M = Ru (**5c**), Os (**6c**)), in which the ligand adopts a *facial* κ^3^ coordination mode, have demonstrated the ability to heterolytically activate molecular hydrogen, behaving as transition-metal frustrated Lewis pairs. These species catalyse the hydrogenation of polar unsaturated bonds, including C=N bonds in *N*-benzylideneaniline and quinoline, C=C bonds in methyl acrylate, and C=O bonds in 2,2,2-trifluoroacetophenone. Notably, both the catalytic performance and stability of these complexes are strongly influenced by the hemilability of the pyridyl–metal bond, which plays a pivotal role in generating the active FLP form. This dynamic coordination behaviour appears to be crucial for efficient hydrogen activation and effective substrate conversion.

## 4. Materials and Methods

All preparations have been carried out under argon. All solvents were treated in a PS-400–6 Innovative Technologies Solvent Purification System (SPS, Innovative Technologies, Wilmington, DE, USA) and degassed before use. Infrared spectra were recorded on a Perkin-Elmer Spectrum-100 (ATR mode) FT-IR spectrometer. Carbon, hydrogen, nitrogen, and sulphur analyses were performed using a Perkin-Elmer 240 B microanalyser (Perkin-Elmer, Shelton, CT, USA). ^1^H and ^13^C NMR spectra were recorded on a Bruker AV-300 spectrometer (300.13 MHz), Bruker AV-400 (400.16 MHz) or Bruker AV-500 (500.13 MHz) (Bruker, Billerica, MA, USA). In both ^1^H NMR and ^13^C NMR measurements, the chemical shifts are expressed in ppm downfield from SiMe_4_. *J* values are given in Hz (the coupling constants are values measured experimentally, accordingly, the difference observed between the values of the constants of the hydrogens in the pyridyl group). NOESY, ^13^C, and ^1^H correlation spectra were obtained using standard procedures. Mass spectra were obtained with a Micro Tof-Q Bruker Daltonics spectrometer (Bruker, Billerica, MA, USA).

### 4.1. Preparation of H_2_NNS

At RT, a mixture of 2-pyridylmethanamine (1000.0 mg, 9.26 mmol) and *p*-tolyl-isothiocyanate (1151.4 μL, 9.26 mmol) in dry THF (10 mL) was stirred for 15 h. During this time, a white solid precipitated, and the THF was removed by vacuum filtration. The resulting white solid was washed with *n*-hexane (3 × 5 mL) and vacuum-dried.


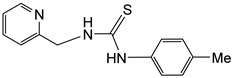


**H_2_NNS**. Yield: 2268 mg, 95%. Anal. Calcd for C_14_H_15_N_3_S: C, 65.34; H, 5.87; N, 16.33; S, 12.46. Found: C, 65.20; H, 5.79; N, 16.20; S, 12.34. HRMS (*μ*-TOF), C_14_H_16_N_3_S, [M + H]^+^, calcd: 258.1059, found: 258.1068. IR (cm^−1^): *ν*(NH) 3323 (m). ^1^H NMR (500.10 MHz, CD_2_Cl_2_, RT): *δ* = 8.44 (bs, 1H, H_6_ Py), 8.03 (bs, 1H, N*Hp*-Tol), 7.69 (t, *J* = 7.8 Hz, 1H, H_4_ Py), 7.65 (bs, 1H, N*H*CH_2_), 7.31 (d, *J* = 7.8 Hz, 1H, H_3_ Py), 7.26 (d, *J* = 8.3 Hz, 2H, CH *p*-Tol), 7.22 (d, 2H, CH *p*-Tol), 7.21 (t, *J* = 7.5 Hz, 1H, H_5_ Py), 4.89 (bs, 2H, CH_2_) and 2.38 (s, 3H, Me). ^13^C{^1^H} NMR (125.77 MHz, CD_2_Cl_2_, RT): *δ* = 181.09 (C=S), 156.60 (C_2_ Py), 149.54 (C_6_ Py), 137.54 (C_4_ Py), 136.55, 133.89, 131.22, 125.50 (CH *p-*Tol), 123.19 (C_5_ Py), 122.71 (C_3_ Py), 50.60 (CH_2_) and 21.54 (Me).

### 4.2. Preparation of the Complexes [(Cym)MCl(κ^2^N_py_,S-H_2_NNS)][SbF_6_] (M = Ru (***1***), Os (***2***)

To a suspension of the corresponding dimer [{(Cym)MCl}_2_(*μ-*Cl)_2_] (0.5 mmol) in methanol (10 mL), 257.3 mg (1.0 mmol) of the **H_2_NNS** ligand and 258.7 mg (1.0 mmol) of NaSbF_6_ were added. The resulting solution was stirred for 5 h and vacuum-evaporated to dryness. The residue was extracted with dichloromethane, and the solution was concentrated under reduced pressure to ca. 2 mL. The slow addition of *n*-hexane led to the precipitation of orange (**1**) or yellow-brown (**2**) solids, which were washed with *n*-hexane (3 × 10 mL) and vacuum-dried.


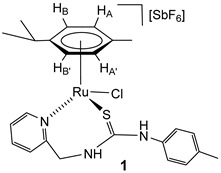


**Complex 1.** Yield: 733 mg, 96%. Anal. Calcd for C_24_H_29_N_3_ClF_6_RuSSb: C, 37.74; H, 3.83; N, 5.50; S, 4.20. Found: C, 38.11; H, 3.78; N, 5.69; S, 4.27. HRMS (*μ*-TOF), C_24_H_29_N_3_ClRuS, [M − SbF_6_]^+^, calcd: 528.0809, found: 528.0790. IR (cm^−1^): *ν*(NH) 3340–3195 (br), *ν*(SbF_6_) 651 (s). ^1^H NMR (500.10 MHz, (CD_3_)_2_CO, RT): *δ* = 9.81 (s, 1H, N*Hp*-Tol), 9.52 (d, *J* = 5.8 Hz, 1H, H_6_ Py), 8.36 (bt,1H, N*H*CH_2_), 8.06, (td, *J* = 7.6, *J* = 1.6 Hz, 1H, H_4_ Py), 7.62 (dd, *J* = 7.4 Hz, *J* = 1.4, 1H, H_3_ Py), 7.61 (td, *J* = 7.5, *J* = 1.5 Hz 1H, H_5_ Py), 7.25 (d, *J* = 8.2 Hz, 2H, C*H*CMe *p*-Tol), 7.16 (dd, *J* = 1.6 Hz, 2H, CHCN *p*-Tol), 5.96 (d, 2H, H_B_, H_B’_), 5.80 (d, *J* = 6.1 Hz, 1H, H_A_), 5.68 (d, *J* = 6.1 Hz, 1H, H_A’_), 5.69 (overlapped, C*H*H), 4.79 (dd, *J* = 14.4 Hz, *J* = 4.9 Hz, 1H, CH*H*), 3.01 (sp, 1H, CH *i*Pr), 2.35 (s, 3H, Me *p*-Tol), 1.92 (s, 3H, Me Cym), 1.36 and 1.35 (2 × d, *J* = 6.9 Hz, 6H, Me *i*Pr).^13^C NMR (125.77 MHz, (CD_3_)_2_CO, RT): *δ* = 178.49 (C=S), 160.50 (C_2_ Py), 160.37 (C_6_ Py), 141.34 (C_4_ Py), 131.98 (*C*HCMe *p*-Tol), 127.97 (C_3_ Py), 126.91 (*C*HCN *p*-Tol), 126.42 (C_5_ Py), 105.71 (*Ci*Pr), 102.99 (*C*Me Cym), 90.00 (CH_B_), 787.25 (CH_A’_, CH_B’_), 85.53 (CH_A’_), 53.24 (CH_2_), 32.05 (*C*H *i*Pr), 23.14, 23.10 (Me *i*Pr), 21.69 (Me *p*-Tol) and 18.57 (Me Cym).


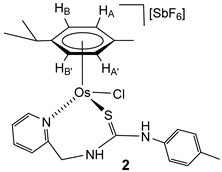


**Complex 2.** Yield: 768 mg, 90%. Anal. Calcd for C_24_H_29_N_3_ClF_6_OsSSb: C, 33.8; H, 3.4; N, 4.95; S, 3.8. Found: C, 33.7; H, 3.7; N, 5.0; S, 4.0. HRMS (*μ*-TOF), C_24_H_28_N_3_OsS, [M − H − Cl − SbF_6_]^+^, calcd: 582.1612, found: 582.1622. IR (cm^−1^): *ν*(NH) 3400–3000 (br), *ν*(SbF_6_) 653 (s). ^1^H NMR (500.10 MHz, (CD_3_)_2_CO, RT (NH resonances at −80 °C)): *δ* = 9.70 (s, 1H, N*Hp*-Tol), 9.42 (d, *J* = 6.0 Hz, 1H, H_6_ Py), 8.30 (bt,1H, N*H*CH_2_), 8.00, (t, *J* = 7.5 Hz, 1H, H_4_ Py), 7.63 (d, *J* = 7.5 Hz, 1H, H_3_ Py), 7.56 (t, *J* = 7.1 Hz 1H, H_5_ Py), 7.25, 7.20 (2 × d, *J* = 8.4 Hz, 4H, CH *p-*Tol), 6.21 (d, *J* = 5.8 Hz, 2H, H_B_, H_B’_), 6.12 (d,1H, H_A’_), 5.87 (d,1H, H_A_), 5.65 (d, *J* = 14.5 Hz, 1H, CH*H_pro-S_*), 4.74 (d, 1H, CH*H_pro-R_*), 2.89 (spt, 1H, CH *i*Pr), 2.35 (s, 3H, Me *p*-Tol), 1.92 (s, 3H, Me Cym), 1.36 and 1.32 (2 × d, *J* = 6.8 Hz, 6H, Me *i*Pr).^13^C NMR (125.77 MHz, (CD_3_)_2_CO, RT): *δ* = 177.92 (C=S), 161.06 (C_6_ Py), 159.43 (C_2_ Py), 141.45 (C_4_ Py), 139.00, 134.76, 131.84 (CH *p*-Tol), 127.60 (C_3_ Py), 126.81 (CH *p*-Tol), 126.73 (C_5_ Py), 96.67 (*C*Me Cym), 94.85 (*Ci*Pr), 82.51 (CH_B_), 78.98 (CH_A_), (CH_B’_), 77.04 (CH_A’_), 53.20 (CH_2_), 32.06 (CH *i*Pr), 23.39, 23.28 (Me *i*Pr), 21.68 (Me *p*-Tol) and 18.37 (Me Cym).

### 4.3. Preparation of the Complexes ***3***

To a solution of the complex [(Cym)RuCl(κ^2^*N*_py_,*S***-H_2_NNS**)][SbF_6_] (**1**) (550.0 mg, 0.720 mmol) in 10 mL of acetone was added 259.3 mg (0.756 mmol) of AgSbF_6_. The resulting suspension was stirred for 2 h. The AgCl formed was separated via cannula, and the filtrate was concentrated under pressure to *ca*. 2 mL. The slow addition of *n*-hexane led to the precipitation of an orange solid, which was washed with *n*-hexane (3 × 10 mL) and vacuum-dried. A mixture of [(Cym)Ru(H_2_O)(κ^2^*N*_py_,*S***-H_2_NNS**)][SbF_6_]_2_ (**3a**, 39%), [(Cym)Ru(κ^3^*N*_py_,*N*_amine_,*S***-H_2_NNS**)][SbF_6_]_2_ (**3b**, 42%), **3c** (7%), **3d** (6%) and **3e** (6%), was obtained. Addition of 100 μL acetonitrile to 15 mg of the solid obtained dissolved in (CD_3_)_2_CO results in the formation of the **3f** complex.

*Preparation of [(Cym)Ru(NCMe)(κ^2^N_py_,S-**H_2_NNS**)][SbF_6_]_2_ (**3f**).* To a solution of the complex [(Cym)RuCl(*κ*^2^*N*_py_*,S*-**H_2_NNS**)][SbF_6_] (**1**) (75.0 mg, 0.098 mmol) in 10 mL of NCMe was added 33.7 mg (0.098 mmol) of AgSbF_6_. The resulting suspension was stirred for 2 h. The AgCl formed was separated with a cannula, and the filtrate was concentrated under reduced pressure to *ca*. 2 mL. The slow addition of *n*-hexane led to the precipitation of a yellow solid, which was washed with *n*-hexane (3 × 5 mL) and vacuum-dried.


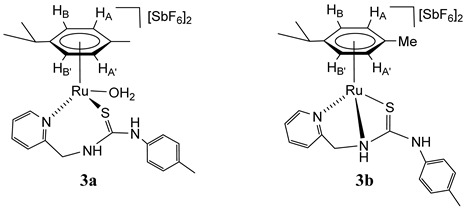


**Complex 3.** Yield: 640 mg, 92% (assuming the solid as **3b**). HRMS (*μ*-TOF), C_24_H_28_N_3_RuS, [M − H − 2 SbF_6_]^+^ and [M − H_2_O − H − 2 SbF_6_]^+^, calcd: 492.1042, found: 492.1059. IR (cm^−1^): *ν*(NH) 3630–3090 (br), *ν*(SbF_6_) 652 (s). **3a**. ^1^H NMR (500.10 MHz, (CD_3_)_2_CO, RT): *δ* = 9.12 (d, *J* = 5.7 Hz, 1H, H_6_ Py), 8.91 (t, *J* = 7.9 Hz, 1H, H_4_ Py), 8.31 (t, *J* = 6.8 Hz, 1H, H_5_ Py), 8.26 (overlapped, 1H, H_3_ Py), 7.47 (d, *J* = 8.5 Hz, 2H, CHCN *p*-Tol), 7.39 (d, 2H, C*H*CMe *p*-Tol), 6.41 (d, *J* = 6.0 Hz, 1H, H_B_), 6.02 (d, *J* = 6.0 Hz, 2H, H_A_, H_B’_), 5.82 (d, 1H, H_A’_), 5.38 (d, *J* = 16.5 Hz, 1H, CH*H_pro-S_*), 4.81 (d, 1H, CH*H_pro-R_*), 2.94 (sp, 1H, CH *i*Pr), 2.39 (s, 3H, Me *p*-Tol), 2.28 (s, 3H, Me Cym), 1.35 and 1.34 (2 × d, *J* = 6.9 Hz, 6H, Me *i*Pr). ^13^C NMR (125.77 MHz, THF-*d*_8_, RT): *δ* = 169.03 (C=S), 150.28 (C_4_ Py), 144.01 (C_6_ Py), 142.65 (C_3_ Py), 143.72 (CN *p*-Tol), 139.64 (*C*Me *p*-Tol), 132.08 (*C*HCMe *p*-Tol), 129.00 (C_5_ Py), 124.38 (*C*HCN *p*-Tol), 109.43 (*Ci*Pr), 105.86 (*C*Me Cym), 86.23, 85.24 (CH_A_, CH_B’_), 86.08 (CH_A’_), 86.06 (CH_B_), 44.25 (CH_2_), 33.03 (CH *i*Pr), 24.11, 23.28 (Me *i*Pr), 21.87 (Me *p*-Tol) and 19.45 (Me Cym). **3b.** ^1^H NMR (500.10 MHz, (CD_3_)_2_CO, RT): *δ* = 9.51 (d, *J* = 5.7 Hz, 1H, H_6_ Py), 8.23 (td, *J* = 7.7 Hz, *J* = 1.4 Hz, 1H, H_4_ Py), 7.87 (d, *J* = 7.7 Hz 1H, H_3_ Py), 7.78 (t, *J* = 6.2 Hz, 1H, H_5_ Py), 7.28 (d, *J* = 8.3 Hz, 2H, C*H*CMe *p*-Tol), 7.11 (d, 2H, CHCN *p*-Tol), 6.45 (d, *J* = 6.2 Hz, 1H, H_B_), 6.38 (d, *J* = 6.2 Hz, 1H, H_A’_), 6.28 (d, 1H, H_B’_), 6.17 (d,1H, H_A_), 5.25 (bs, 2H, CH_2_), 2.94 (sp, 1H, CH *i*Pr), 2.37 (s, 3H, Me Cym), 2.35 (s, 3H, Me *p*-Tol), 1.31 and 1.25 (2 × d, *J* = 6.9 Hz, 6H, Me *i*Pr). ^13^C NMR (125.77 MHz, THF-*d*_8_, RT): *δ* = 193.66 (C=S), 156.82 (C_6_ Py), 142.09 (C_4_ Py), 140.09 (*C*Me *p*-Tol), 138.86 (CN *p*-Tol), 132.08 (*C*HCMe *p*-Tol), 132.21 (*C*HCMe *p*-Tol), 128.11 (C_5_ Py), 127.08 (*C*HCN *p*-Tol), 125.94 (C_3_ Py), 110.48 (*Ci*Pr), 103.47 (*C*Me Cym), 86.79 (CH_A_), 86.70 (CH_B’_), 85.96 (CH_A’_), 85.24 (CH_B_), 61.40 (CH_2_), 32.96 (CH *i*Pr), 23.53, 23.06 (Me *i*Pr), 21.77 (Me *p*-Tol) and 19.64 (Me Cym). **3c.** ^1^H NMR (500.10 MHz, (CD_3_)_2_CO, RT): *δ* = 9.37 (d, *J* = 5.5 Hz, 1H, H_6_ Py), 8.05 (td, *J* = 7.7 Hz, *J* = 1.4 Hz, 1H, H_4_ Py), 7.66 (d, *J* = 7.7 Hz, 1H, H_3_ Py), 7.57 (t, *J* = 6.5 Hz, 1H, H_5_ Py), 7.36 (*J* = 8.0 Hz, 2H, CHCN *p*-Tol), 7.29 (d, 2H, C*H*CMe *p*-Tol), 6.22 (d, *J* = 6.1 Hz, 1H, H_B_), 5.95 (d, *J* = 6.4 Hz, H_A’_), 5.87 (d, 2H, H_B’_), 5.66 (d, 1H, H_A_), 5.46, 5.27 (d, *J* = 14.3 Hz, 2H, CH_2_), 3.26 (sp, 1H, CH *i*Pr), 2.65 (s, 3H, Me Cym), 2.42 (s, 3H, Me *p*-Tol), 1.59 and 1.51 (2 × d, *J* = 6.9 Hz, 6H, Me *i*Pr). ^13^C NMR (125.77 MHz, THF-*d*_8_, RT): *δ* = 163.90 (C=S), 157.39 (C_6_ Py), 141.69 (C_4_ Py), 127.42 (C_5_ Py), 123.52 (C_3_ Py), 138.86 (CN *p*-Tol), 137.8 (*C*Me *p*-Tol), 130.45 (*C*HCMe *p*-Tol), 128.68 (*C*HCN *p*-Tol), 109.40 (*Ci*Pr), 106.49 (*C*Me Cym), 92.01 (CH_B_), 91.71 (CH_A’_), 90.85 (CH_B’_), 89.20 (CH_A_), 63.08 (CH_2_), 32.90 (CH *i*Pr), 23.84, 23.78 (Me *i*Pr) and 19.31 (Me Cym). **3d**. ^1^H NMR (500.10 MHz, (CD_3_)_2_CO, RT): *δ* = 3.06 (m, 1H, CH *i*Pr), 1.40 (bd, *J* = 6.3 Hz, 6H, Me *i*Pr). **3e**. ^1^H NMR (500.10 MHz, (CD_3_)_2_CO, RT): *δ* = 2.75 (sp, 1H, CH *i*Pr), 1.22 and 0.89 (2 × d, *J* = 6.9 Hz, 6H, Me *i*Pr).


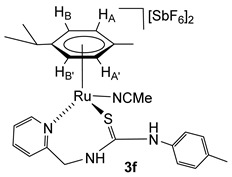


**Complex 3f.** Yield: 74 mg, 75%. Anal. Calcd for C_26_H_32_N_4_F_12_RuSSb_2_: C, 31.07; H, 3.21; N, 5.57; S, 3.19. Found: C, 31.47; H, 3.36; N, 5.84; S, 3.17. HRMS (*μ*-TOF), C_24_H_29_N_3_RuS, [M − 2 SbF_6_ − NCMe]^2+^, calcd: 246.5557, found: 246.5551. IR (cm^−1^): *ν*(NH) 3334 (br), *ν*(SbF_6_) 652 (s). ^1^H NMR (400.16 MHz, CD_2_Cl_2_, RT): *δ* = 9.17 (d, *J* = 6.0 Hz, 1H, H_6_ Py), 8.22 (s, 1H, N*Hp*-Tol), 8.04 (td, *J* = 7.7 Hz, *J* = 1.4 Hz, 1H, H_4_ Py), 7.71 (d, *J* = 6.4 Hz, 1H, H_3_ Py), 7.68 (td, *J* = 7.7 Hz, *J* = 1.6 Hz, 1H, H_5_ Py), 7.27 (d, *J* = 8.2 Hz, 2H, C*H*CMe *p*-Tol), 7.08 (d, 2H, CHCN *p*-Tol), 7.13 (bs, 1H, N*H*CH_2_), 6.11 (d, *J* = 6.2 Hz, 1H, H_B_), 6.00 (d, *J* = 6.1 Hz, 1H, H_B’_) 5.85 (d,1H, H_A_), 5.64 (d, 1H, H_A’_), 5.18 (dd, *J* = 15.1 Hz, *J* = 7.9 Hz, 1H, CH*H_pro-S_*), 4.62 (dd, *J* = 5.6 Hz, 1H, CH*H_pro-R_*), 2.82 (m, 1H, CH *i*Pr), 2.56 (s, 3H, NCMe), 2.36 (s, 3H, Me *p*-Tol), 1.80 (s, 3H, Me Cym), 1.32 and 1.28 (2 × d, *J* = 7.1 Hz, 6H, Me *i*Pr). ^13^C NMR (125.77 MHz, CD_2_Cl_2_, RT): *δ* = 176.88 (C=S), 158.47 (C_2_ Py), 158.27 (C_6_ Py), 142.25 (C_4_ Py), 131.82 (*C*HCMe *p*-Tol), 128.80 (C_3_ Py), 128.58 (N*C*Me), 127.76 (C_5_ Py), 126.07 (*C*HCN *p*-Tol), 108.46 (*Ci*Pr), 107.47 (*C*Me Cym), 90.75 (CH_B’_), 89.20 (CH_A_), 88.69 (CH_B_), 85.03 (CH_A’_), 52.68 (CH_2_), 31.46 (CH *i*Pr), 23.17, 22.21 (Me *i*Pr), 21.63 (Me *p*-Tol), 18.45 (Me Cym) and 4.75 (NC*Me*).

### 4.4. Preparation of the Complex [(Cym)Os(NCMe)(κ^2^N_py_,S-H_2_NNS)][SbF_6_]_2_ (***4a***)

To a solution of the complex [(Cym)OsCl(*κ*^2^*N*_py_*,S*-**H_2_NNS**)][SbF_6_] (**2**) (300.0 mg, 0.352 mmol) in 10 mL of NCMe was added 120.8 mg (0.352 mmol) of AgSbF_6_. The resulting suspension was stirred for 2 h. The AgCl formed was separated with a cannula, and the filtrate was concentrated under reduced pressure to *ca*. 2 mL. The slow addition of *n*-hexane led to the precipitation of a yellow solid, which was washed with *n*-hexane (3 × 5 mL) and vacuum-dried.


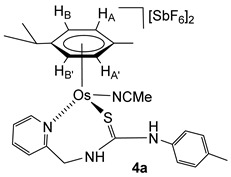


**Complex 4a.** Yield: 289 mg, 75%. Anal. Calcd for C_26_H_32_N_4_F_12_OsSSb_2_: C, 28.5; H, 2.9; N, 5.1; S, 2.9. Found: C, 28.4; H, 3.0; N, 5.0; S, 3.2. HRMS (*μ*-TOF), C_24_H_28_N_3_OsS, [M − 2 SbF_6_ − NCMe − H]^+^, calcd: 582.1612, found: 582.1635. IR (cm^−1^): *ν*(NH) 3335 (br), *ν*(SbF_6_) 651 (s). ^1^H NMR (500.10 MHz, CD_2_Cl_2_, RT): *δ* = 9.12 (d, *J* = 6.1 Hz, 1H, H_6_ Py), 8.16 (s, 1H, N*Hp*-Tol), 8.00 (t, *J* = 7.7 Hz, 1H, H_4_ Py), 7.71 (d, *J* = 7.7 Hz, 1H, H_3_ Py), 7.63 (t, *J* = 6.9 Hz, 1H, H_5_ Py), 7.24 (bt, *J* = 9.3 Hz, 1H, N*H*CH_2_), 7.29 (d, *J* = 8.2 Hz, 2H, C*H*CMe *p*-Tol), 7.10 (d, 2H, CHCN *p*-Tol), 6.37 (d, *J* = 5.8 Hz, 1H, H_B_), 6.14 (d, *J* = 5.8 Hz, 1H, H_B’_) 5.93 (d,1H, H_A_), 5.88 (d, 1H, H_A’_), 5.27 (dd, *J* = 14.8 Hz, *J* = 8.0 Hz, 1H, CH*H_pro-S_*), 4.58 (dd, *J* = 5.7 Hz, 1H, CH*H_pro-R_*), 2.81 (s, 3H, NCMe), 2.77 (m, 1H, C*H i*Pr), 2.37 (s, 3H, Me *p*-Tol), 1.83 (s, 3H, Me Cym), 1.31 and 1.28 (2 × d, *J* = 7.0 Hz, 6H, Me *i*Pr). ^13^C NMR (125.77 MHz, CD_2_Cl_2_, RT): *δ* = 175.72 (C=S), 159.23 (C_6_ Py), 157.63 (C_2_ Py), 142.61 (C_4_ Py), 141.80 (CN *p*-Tol), 140.74 (*C*Me *p*-Tol), 131.88 (*C*HCMe *p*-Tol), 128.45 (C_3_ Py), 128.24 (C_5_ Py), 126.15 (*C*HCN *p*-Tol), 124.15 (N*C*Me), 99.64 (*Ci*Pr), 99.01 (*C*Me Cym), 83.78 (CH_B’_), 81.86 (CH_B_), 81.38 (CH_A_), 77.15 (CH_A’_), 52.79 (CH_2_), 23.29 (CH *i*Pr), 22.60, 21.65 (Me *i*Pr), 20.87 (Me *p*-Tol), 18.10 (Me Cym) and 4.71 (NC*Me*).

### 4.5. Preparation of the Complexes ***5***

To a solution of the complex **3** (400 mg, 0.415 mmol, assuming the solid as **3b**) in methanol (20 mL), 34.9 mg (0.415 mmol) of solid NaHCO_3_ was added. The resulting suspension was stirred for 10 h, filtered to remove a small fraction of a dark solid in suspension, and the resulting solution was evaporated to dryness. The residue was extracted with dichloromethane, and the resulting solution was concentrated under reduced pressure to *ca*. 2 mL. The slow addition of *n*-pentane led to the precipitation of a yellow-brown solid, which was washed with *n*-pentane (3 × 5 mL) and vacuum-dried. A mixture of [(Cym)Ru(κ^3^*N*_py,_*N*_amide_,*S***-H_2_NNS**)][SbF_6_] (**5a**, 42%), [(Cym)Ru(H_2_O)(κ^2^*N*_amide_,*S***-H_2_NNS**)][SbF_6_] (**5b**, 38%) and [(Cym)Ru(κ^3^*N*_py,_*N*_amide_,*S***-H_2_NNS**)][SbF_6_] (**5c**, 20%) was obtained. Addition of 0.1 mL of D_2_O and H_2_ (5 bar) to 15 mg of the solid obtained dissolved in THF-*d*_8_ (0.35 mL) results in the formation of **5c** complex in 5 days at RT.

In an NMR tube 15 mg of a mixture of [(Cym)Ru(κ^3^*N*_py,_*N*_amide_,*S***-HNNS**)][SbF_6_] (**5a**, 42%), [(Cym)Ru(H_2_O)(κ^2^*N*_amide_,*S***-HNNS**)][SbF_6_] (**5b**, 38%) and [(Cym)Ru(κ^3^*N*_py,_*N*_amide_,*S*-**HNNS**)][SbF_6_] (**5c**, 20%) was heated in methanol at 50 °C for 5 h. The ^1^H NMR spectrum showed the presence of a 27/24/49 molar ratio of compounds **5a**/**5b**/**5c** together with free *p*-cymene (13%).

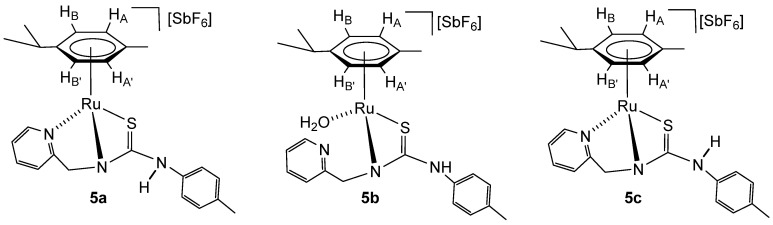



**Complex 5.** Yield: 242 mg, 80% (assuming the solid as **5c**). Anal. Calcd for C_24_H_28_N_3_F_6_RuSSb: C, 39.63; H, 3.88; N, 5.78; S, 4.41. Found: C, 40.04; H, 3.64; N, 5.94; S, 4.37. HRMS (*μ*-TOF), C_24_H_28_N_3_RuS, [M − SbF_6_]^+^, calcd: 492.1042, found: 492.1066. IR (cm^−1^): *ν*(NH) 3368 (br), *ν*(SbF_6_) 653 (s). **5a**. ^1^H NMR (500.10 MHz, THF-*d*_8_, RT): *δ* = 9.28 (bs, 1H, H_6_ Py), 8.83 (s, 1H, NH), 7.92 (m,1H, H_4_ Py), 7.60 (t, *J* = 6.5 Hz, 1H, H_5_ Py), 7.54 (m, 1H, H_3_ Py), 7.46 (dd, *J* = 8.5 Hz, *J* = 1.9 Hz, 2H, CHCN *p*-Tol), 7.33 (d, 2H, C*H*CMe *p*-Tol), 6.11(d, *J* = 6.2 Hz, 1H, H_B_), 5.87 (d, *J* = 5.9 Hz, 1H, H_A’_), 5.65 (d, 1H, H_B’_), 5.28 (d,1H, H_A_), 5.14 (d, *J* = 19.5 Hz, 1H, CH*H_pro-S_*), 4.76 (d, 1H, CH*H_pro-R_*), 3.07 (sp, 1H, CH *i*Pr), 2.59 (s, 3H, Me Cym), 2.47 (s, 3H, Me *p*-Tol), 1.52 and 1.39 (2 × d, *J* = 7.1 Hz, 6H, Me *i*Pr). ^13^C NMR (125.77 MHz, THF-*d*_8_, RT): *δ* = 163.33 (C=S), 156.52 (C_6_ Py), 140.57 (C_4_ Py), 138.37 (CN *p*-Tol), 136.95 (*C*Me *p*-Tol), 129.56 (*C*HCMe *p*-Tol), 128.31, 128.26 (*C*HCN *p*-Tol), 126.21 (C_5_ Py), 122.73 (C_3_ Py), 108.32 (*Ci*Pr), 105.80 (*C*Me Cym), 92.09 (CH_B_), 90.10 (CH_B’_), 89.51 (CH_A_), 89.43 (CH_A’_), 62.21 (CH_2_), 32.34 (*C*H *i*Pr), 23.07, 22.87 (Me *i*Pr), 21.15 (Me *p*-Tol) and 18.37 (Me Cym). **5b**. ^1^H NMR (500.10 MHz, THF-*d*_8_, RT): *δ* = 8.59 (bs, 1H, H_6_ Py), 7.71 (t, *J* = 7.3 Hz, 1H, H_4_ Py), 7.29 (t, *J* = 6.0 Hz, 1H, H_5_ Py), 7.08 (overlapped, 1H, H_3_ Py), 7.09 (d, *J* = 8.0 Hz, 2H, C*H*CMe *p*-Tol), 7.02 (d, 2H, CHCN *p*-Tol), 5.42 (d, *J* = 6.1 Hz, 1H, H_B_), 5.41 (d, *J* = 6.0 Hz, 1H, H_A’_), 5.33 (d,1H, H_A_), 5.22 (d, 1H, H_B’_), 3.64 (bs, 2H, CH_2_), 2.79 (sp, 1H, CH *i*Pr), 2.33 (s, 3H, Me Cym), 2.31 (s, 3H, Me *p*-Tol), 1.22 and 1.03 (2 × d, *J* = 6.9 Hz, 6H, Me *i*Pr). ^13^C NMR (125.77 MHz, THF-*d*_8_, RT): *δ* = 177.55 (C=S), 149.81 (C_6_ Py), 144.68 (CN *p*-Tol), 137.21 (*C*Me *p*-Tol), 137.83 (C_4_ Py), 130.65 (*C*HCMe *p*-Tol), 124.48 (*C*HCN *p*-Tol), 123.73 (C_5_ Py), 122.85 (C_3_ Py), 108.02 (*Ci*Pr), 101.87 (*C*Me Cym), 84.28 (CH_B’_)_,_ 84.00 (CH_A’_), 83.81 (CH_A_), 83.55 (CH_B_), 48.38 (CH_2_), 31.76 (CH *i*Pr), 23.20, 21.84 (Me *i*Pr), 21.01 (Me *p*-Tol) and 18.50 (Me Cym). **5c**. ^1^H NMR (500.10 MHz, THF-*d*_8_, RT): *δ* = 9.05 (d, *J* = 5.7 Hz, 1H, H_6_ Py), 7.94 (td, *J* = 7.8 Hz, *J* = 1.3 Hz, 1H, H_4_ Py), 7.57 (d, *J* = 7.8 Hz, 1H, H_3_ Py), 7.49 (t, *J* = 6.6 Hz, 1H, H_5_ Py), 7.00 (d, *J* = 8.2 Hz, 2H, C*H*CMe *p*-Tol), 6.95 (d, 2H, CHCN *p*-Tol), 5.97 (d, *J* = 5.9 Hz, 1H, H_B_), 5.86 (d, *J* = 5.9 Hz, 1H, H_A’_), 5.74 (d, 1H, H_B’_), 5.59 (d,1H, H_A_), 4.91 (d, *J* = 16.5 Hz, 1H, CH*H_pro-R_*), 4.64 (d, 1H, CH*H_pro-S_*), 2.68 (sp, 1H, CH *i*Pr), 2.20 (s, 3H, Me *p*-Tol), 2.07 (s, 3H, Me Cym), 1.20 and 1.18 (2 × d, *J* = 6.9 Hz, 6H, Me *i*Pr). ^13^C NMR (125.77 MHz, THF-*d*_8_, RT): *δ* = 155.54 (C_6_ Py), 139.56 (C_4_ Py), 135.22 (*C*Me *p*-Tol), 129.55 (*C*HCMe *p*-Tol), 125.48 (C_5_ Py), 122.46 (*C*HCN *p*-Tol), 122.37 (C_3_ Py), 105.90 (*Ci*Pr), 99.87 (*C*Me Cym), 84.45 (CH_B_), 84.19 (CH_A_), 83.90 (CH_A’_), 83.00 (CH_B’_), 61.20 (CH_2_), 31.12 (CH *i*Pr), 22.63, 22.17 (Me *i*Pr), 20.51 (Me *p*-Tol) and 18.41 (Me Cym).

### 4.6. Preparation of the Complexes ***6***

To a solution of the complex [(Cym)OsCl(*κ*^2^*N*_Py_*,S***-H_2_NNS**)][SbF_6_] (**2**) (255.9 mg, 0.30 mmol) in 10 mL of acetone was added to 103.1 mg (0.30 mmol) of AgSbF_6_. The resulting suspension was stirred for 2 h. The AgCl formed was separated with a cannula, and the filtrate was concentrated under pressure to *ca*. 2 mL. The slow addition of *n*-hexane led to the precipitation of a yellow-brown solid, a mixture of complexes **6a**, **6c**, and **4b**, which was vacuum-dried and characterised in situ by ^1^H NMR. Then, 25.2 mg (0.30 mmol) of NaHCO_3_ and 20 mL of methanol were added to the solid obtained. The resulting suspension was stirred for 10 h, and then was vacuum-evaporated to dryness, and the residue was extracted with dichloromethane. The solution was concentrated under pressure to ca. 2 mL. The slow addition of *n*-hexane led to the precipitation of a yellow-brown solid, which was washed with *n*-hexane (3 × 5 mL) and vacuum-dried. A mixture of [(Cym)Os(κ^3^*N*_py,_*N*_amide_,*S***-HNNS**)][SbF_6_] (**6a**, 37%), [(Cym)Os(H_2_O)(κ^2^*N*_amide_,*S***-HNNS**)][SbF_6_]_2_ (**6b**, 39%) and [(Cym)Ru(κ^3^*N*_py,_*N*_amide_,*S*-**HNNS**)][SbF_6_] (**6c**, 24%) was obtained.

In an NMR tube, 15 mg of a mixture of [(Cym)Os(κ^3^*N*_py,_*N*_amide_,*S***-HNNS**)][SbF_6_] (**6a**, 37%), [(Cym)Os(H_2_O)(κ^2^*N*_amide_,*S***-HNNS**)][SbF_6_] (**6b**, 39%) and [(Cym)Os(κ^3^*N*_py,_*N*_amide_,*S*-**HNNS**)][SbF_6_] (**6c**, 24%) was heated in methanol at 60 °C for 24 h. The ^1^H NMR spectrum showed the presence of a 12/16/72 molar ratio of compounds **6a**/**6b**/**6c**.

The lower solubility of compound **6c** in methanol allowed **6c** to be separated as a yellow solid with 98% purity, and from the mother liquors, a 49/49/2 mixture of **6a**/**6b**/**6c** could be isolated.

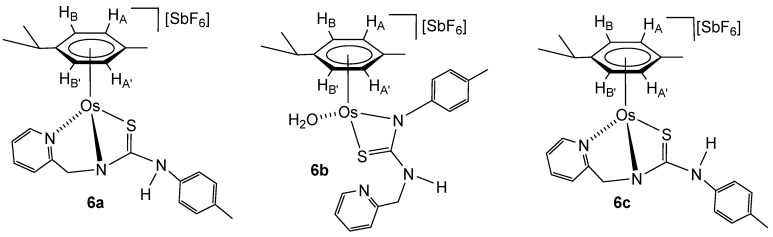



**Complex 6.** Yield: 194 mg, 79% (assuming the solid as **6c**). Anal. Calcd for C_24_H_28_N_3_F_6_OsSSb: C, 35.3; H, 3.5; N, 5.15; S, 3.9. Found: C, 35.5; H, 3.7; N, 5.2; S, 3.9. HRMS (*μ*-TOF), C_24_H_28_N_3_OsS, [M − SbF_6_]^+^, calcd: 582.1612, found: 582.1641. IR (cm^−1^): *ν*(NH) 3600–3000 (br), *ν*(SbF_6_) 653 (s). **6a**. ^1^H NMR (500.10 MHz, CD_2_Cl_2_, RT): *δ* = 9.00 (d, *J* = 5.7 Hz, 1H, H_6_ Py), 7.89 (s, 1H, NH), 7.74 (t, *J* = 7.5 Hz, 1H, H_4_ Py), 7.64 (d, *J* = 7.1 Hz, 1H, H_3_ Py), 7.47 (t, *J* = 6.6 Hz, 1H, H_5_ Py), 7.40, 7.28 (2 × d, *J* = 8.2 Hz, 4H, CH *p*-Tol), 6.01 (d, *J* = 5.8 Hz, 1H, H_B_), 5.26 (d,1H, H_A_), 5.77 (d, *J* = 5.7 Hz, 1H, H_A’_), 5.52 (d, 1H, H_B’_), 5.36 (d, *J* = 18.9 Hz, 1H, CH*H_pro-R_*), 4.90 (d, 1H, CH*H_pro-S_*), 2.87 (sp, 1H, CH *i*Pr), 2.56 (s, 3H, Me Cym), 2.42 (s, 3H, Me *p*-Tol), 1.47, 1.37 (2 × d, *J* = 6.7 Hz, 6H, Me *i*Pr). ^13^C NMR (125.77 MHz, CD_2_Cl_2_, RT): *δ* = 176.50 (C=S), 162.08 (C_2_ Py), 155.22(C_6_ Py), 141.25 (C_4_ Py), 138.26, 136.77, 129.85, 128.14 (CH *p*-Tol), 127.84 (C_5_ Py), 126.29 (C_3_ Py), 99.01 (*C*Me Cym), 98.37 (*Ci*Pr), 84.43 (CH_B_), 82.01 (CH_B’_), 80.96 (CH_A’_), 79.91 (CH_A_), 62.46 (CH_2_), 32.41 (CH *i*Pr), 23.88, 23.09 (Me *i*Pr), 21.58 (Me *p*-Tol) and 19.02 (Me Cym). **6b**. ^1^H NMR (500.10 MHz, CD_2_Cl_2_, RT): *δ* = 8.39 (d, *J* = 5.6 Hz, 1H, H_6_ Py), 8.07 (t, *J* = 7.8 Hz, 1H, H_4_ Py), 7.63 (t, *J* = 7.4 Hz, 1H, H_5_ Py), 7.13 (d, *J* = 4.4 Hz, 1H, H_3_ Py), 6.89, 6.54 (2 × d, *J* = 7.4 Hz, 4H, CH *p*-Tol), 5.47 (d, *J* = 5.6 Hz, 1H, H_A_), 5.45 (d, *J* = 5.7 Hz, 1H, H_A’_), 5.23 (d,1H, H_B’_), 5.19 (d,1H, H_B_), 4.73 (bt, 1H, NH), 3.50, 3.38 (2 × dd, *J* = 17.1 Hz, *J* = 7.0 Hz, 2H, CH_2_), 2.50 (s, 3H, Me Cym), 2.41 (m, 1H, CH *i*Pr), 2.22 (s, 3H, Me *p*-Tol), 1.10 and 0.68 (2 × d, *J* = 7.0 Hz, 6H, Me *i*Pr). ^13^C NMR (125.77 MHz, CD_2_Cl_2_, RT): *δ* = 171.16 (C=S), 151.49 (C_2_ Py), 148.28 (C_6_ Py), 143.38 (CH *p*-Tol), 141.90 (C_4_ Py), 139.11, 130.76 (CH *p*-Tol), 125.06 (C_3_ Py), 123.72 (CH *p*-Tol), 122.51 (C_5_ Py), 97.78 (*C*Me Cym), 93.57 (*Ci*Pr), 77.45 (CH_B’_), 76.32 (CH_B_), 75.37 (CH_A_), 73.55 (CH_A’_), 46.09 (CH_2_), 31.49 (CH *i*Pr), 23.81, 21.25 (Me *i*Pr), 21.41 (Me *p*-Tol) and 19.79 (Me Cym). **6c**. ^1^H NMR (500.10 MHz, CD_2_Cl_2_, RT): *δ* = 8.87 (d, *J* = 5.5 Hz, 1H, H_6_ Py), 7.81 (t, *J* = 7.7 Hz, 1H, H_4_ Py), 7.51 (d, *J* = 7.8 Hz, 1H, H_3_ Py), 7.33 (t, *J* = 6.6 Hz, 1H, H_5_ Py), 7.11, 7.05 (2 × d, *J* = 8.5 Hz, 4H, CH *p*-Tol), 5.95 (d, *J* = 5.4 Hz, 1H, H_B_), 5.75 (bs,1H, NH), 5.57 (d,1H, H_A_), 5.83, 5.81 (2 × d, *J* = 5.8 Hz, 2H, H_A’_, H_B’_), 5.10 (d, *J* = 16.8 Hz, 1H, CH*H_pro-R_*), 4.54 (d, 1H, CH*H_pro-S_*), 2.63 (sp, 1H, CH *i*Pr), 2.29 (s, 3H, Me *p*-Tol), 2.24 (s, 3H, Me Cym), 1.23 and 1.21 (2 × d, *J* = 7.0 Hz, 6H, Me *i*Pr). ^13^C NMR (125.77 MHz, CD_2_Cl_2_, RT): *δ* = 164.02 (C_2_ Py), 155.08 (C_6_ Py), 139.87 (C_4_ Py), 137.29, 137.01, 130.64 (CH *p*-Tol), 125.99 (C_5_ Py), 122.94 (CH *p*-Tol), 122.30 (C_3_ Py), 97.09 (*C*Me Cym), 91.34 (*Ci*Pr), 76.28 (CH_B_), 75.73, 74.57 (CH_A’_, CH_B’_), 74.69 (CH_A_), 62.66 (CH_2_), 32.85 (CH *i*Pr), 23.79, 23.21 (Me *i*Pr), 21.47 (Me *p*-Tol) and 19.79 (Me Cym).

**Complex 4b.** ^1^H NMR (500.10 MHz, CD_3_OD, RT): *δ* = 12.45 (s, 1H, NH pyridinium), 8.35 (bt, *J* = 5.0, 1H, H_6_ Py). Complex **4b** was characterised in situ by ^1^H NMR from a mixture of **6a**, **6c,** and **4b**. All other resonances of **4b** not specified are like the resonances of **6b**.

### 4.7. Preparation of the Complexes ***6a***–***6c*** from ***4a***, and ***4a*** from ***6a***–***6c***

*Preparation of the complexes* ***6a***–***6c** from **4a**.* To a solution of the complex **4a** (49.2 mg, 0.045 mmol) in methanol (5 mL), 3.8 mg (0.0455 mmol) of solid NaHCO_3_ was added. The resulting suspension was stirred for 6 h and evaporated to dryness. The residue was extracted with dichloromethane, and the resulting solution was concentrated under reduced pressure to *ca*. 1 mL. The slow addition of *n*-pentane led to the precipitation of a yellow-brown solid (26 mg, Yield 70%), which was washed with *n*-pentane (3 × 3 mL) and vacuum-dried. According to NMR measurements, the solid consists of a 35/36/29, **6a**/**6b**/**6c** mixture.

*Preparation of the complexes* ***4a****from **6a***–***6c***. To a solution of a mixture of complexes **6a**–**6c** (50 mg, 0.047 mmol) in acetonitrile (5 mL) was added HSbF_6_ (3.83 μL, *ρ* = 2.88 g·mL^−1^, 0.047 mmol). The resulting solution was stirred for 5 h and concentrated under reduced pressure to ca. 1 mL. The slow addition of *n*-pentane led to the precipitation of a yellow solid (39 mg, yield 75%), which was washed with pentane (3 × 3 mL) and vacuum-dried.

### 4.8. General Procedure for the Catalytic Hydrogenation Reactions

A high-pressure NMR tube containing the catalyst (0.015 mmol) and the substrate to be hydrogenated (0.30 mmol) in THF-*d*_8_ (0.45 mL) was pressurised with hydrogen gas (5 bar). The tube was heated at the appropriate temperature, and the solution was monitored by NMR. Conversions were determined by ^1^H NMR.

### 4.9. Reaction of the Complexes ***5*** and ***6c*** with H_2_ in the Presence of D_2_O

A high-pressure NMR tube containing a solution of the corresponding complexes **5** or **6c** (0.015 mmol) in THF-*d*_8_/D_2_O (0.35 mL/0.1 mL) was pressurised with H_2_ (5 bar), and the resulting solution was monitored by NMR spectroscopy. After 30 min (complexes **5**) or 5 days (complex **6c**) at RT, HD is formed in the reaction medium.

## Figures and Tables

**Table 1:** Hydrogenation of unsaturated substrates ^1^.

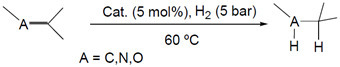					
Entry.	Catalyst	Substrate	Product	t (h)	Conv. (%) ^2^
1	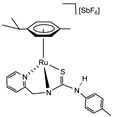 **5c**	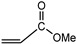	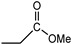	115	97
2		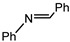	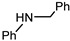	60	97
3		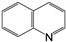	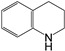	207	97
4		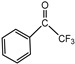	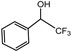	190	53
5	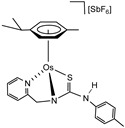 **6c**	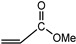	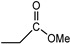	115	9
6		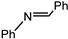	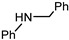	128	97
7		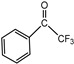	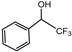	190	22

^1^ Reaction conditions: catalyst 0.015 mmol (5 mol %), substrate (0.30 mmol), H_2_ (5 bar), 0.45 mL of THF-*d*_8_. ^2^ Determined by NMR.

## Data Availability

Data are contained within the article and Supplementary Materials.

## Supplementary Materials

The following supporting information can be downloaded at: https://www.mdpi.com/article/10.3390/molecules30163398/s1, Figure S1: 1H NMR of H2NNS (500.10 MHz, CD2Cl2, RT) to Figure S22: 13C{1H} NMR of [(Cym)Os(κ3Npy,Namide,S-HNNS)][SbF6] (6c) (125.77 MHz, CD2Cl2, RT).
