# Supplementary material for: Pyridyl-Thiourea Ruthenium and Osmium Complexes: Coordination of Ligand and Application as FLP Hydrogenation Catalysts
Source: Molecules. 2025 Aug 16;30(16):3398. doi: 10.3390/molecules30163398 (PMC12388224; doi:10.3390/molecules30163398)
Supplement: Supplementary file 1 [file molecules-30-03398-s001.zip › molecules-3810394-supplementary.pdf]

molecules

Supplementary Materials

Pyridyl-Thiourea Ruthenium and Osmium Complexes: Coordination of Ligand and Application as FLP Hydrogenation Catalysts

Table of Contents

1 NMR spectra for the ligand H_2NNS and the complexes 1-6 **S1**

1 NMR spectra for the ligand H_2NNS and the complexes 1-6

* Denotes solvent

Figure S1. **^{1}H NMR of H_2NNS (500.10 MHz, CD_2Cl_2, RT)**

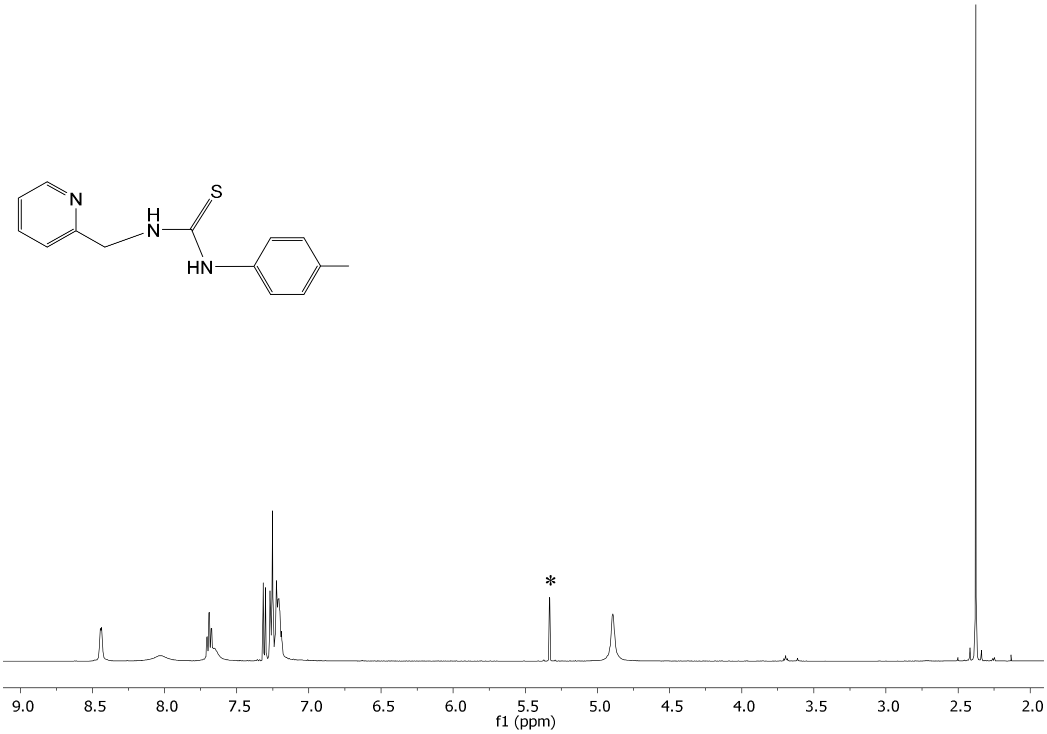

Figure S2. **$^{13}C\{^1H\}$ NMR of H_2NNS (125.77 MHz, CD_2Cl_2, RT)**

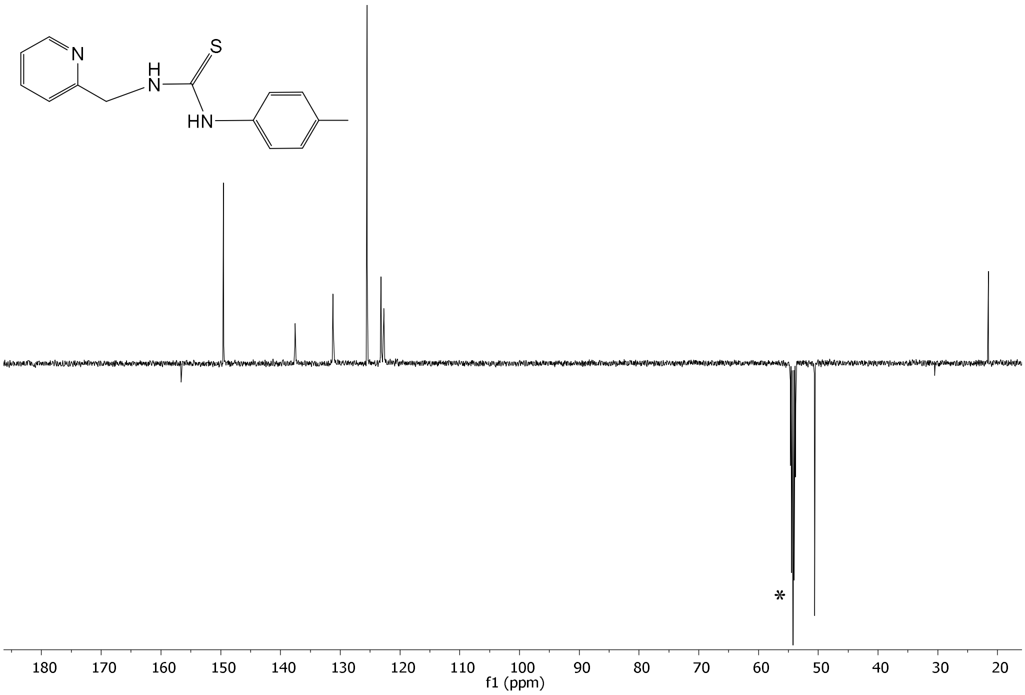

Figure S3. ^{1}H **NMR of [(Cym)RuCl(κ^{2}*N*$_{py}$,*S*-H$_{2}$NNS)][SbF$_{6}$] (1) (500.10 MHz, (CD$_{3}$)$_{2}$CO, RT)**

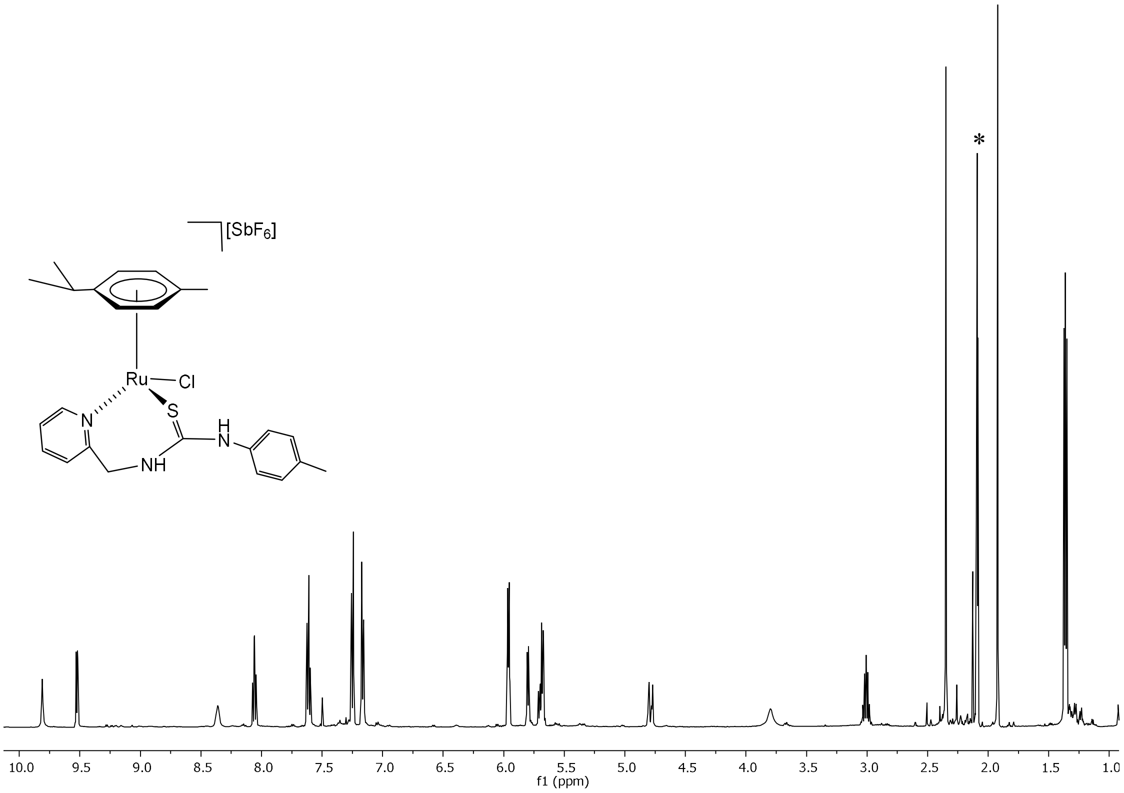

Figure S4. $^{13}C\{^{1}H\}$ **NMR of [(Cym)RuCl(κ^{2}*N*$_{py}$,*S*-H$_{2}$NNS)][SbF$_{6}$] (1) (125.77 MHz, (CD$_{3}$)$_{2}$CO, RT)**

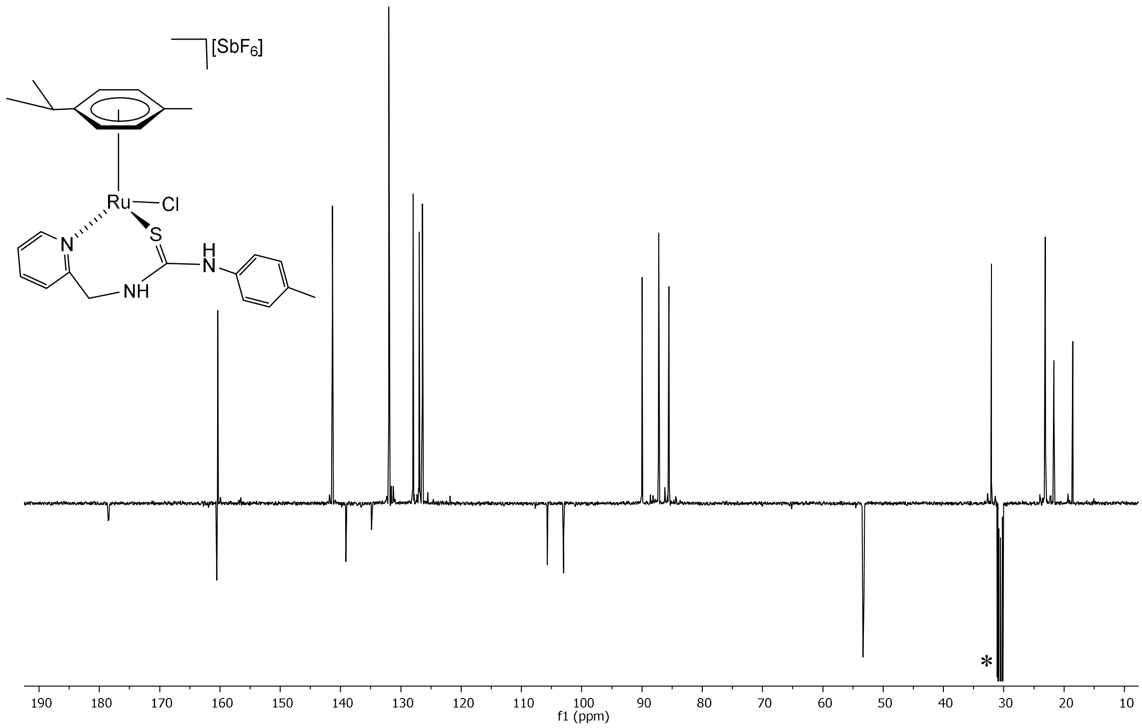

Figure S5. ^{1}H NMR of [(Cym)OsCl(κ^{2}N_{py},S-H_2NNS)][SbF_6] (2) (500.10 MHz, $(CD_3)_2CO$, RT)

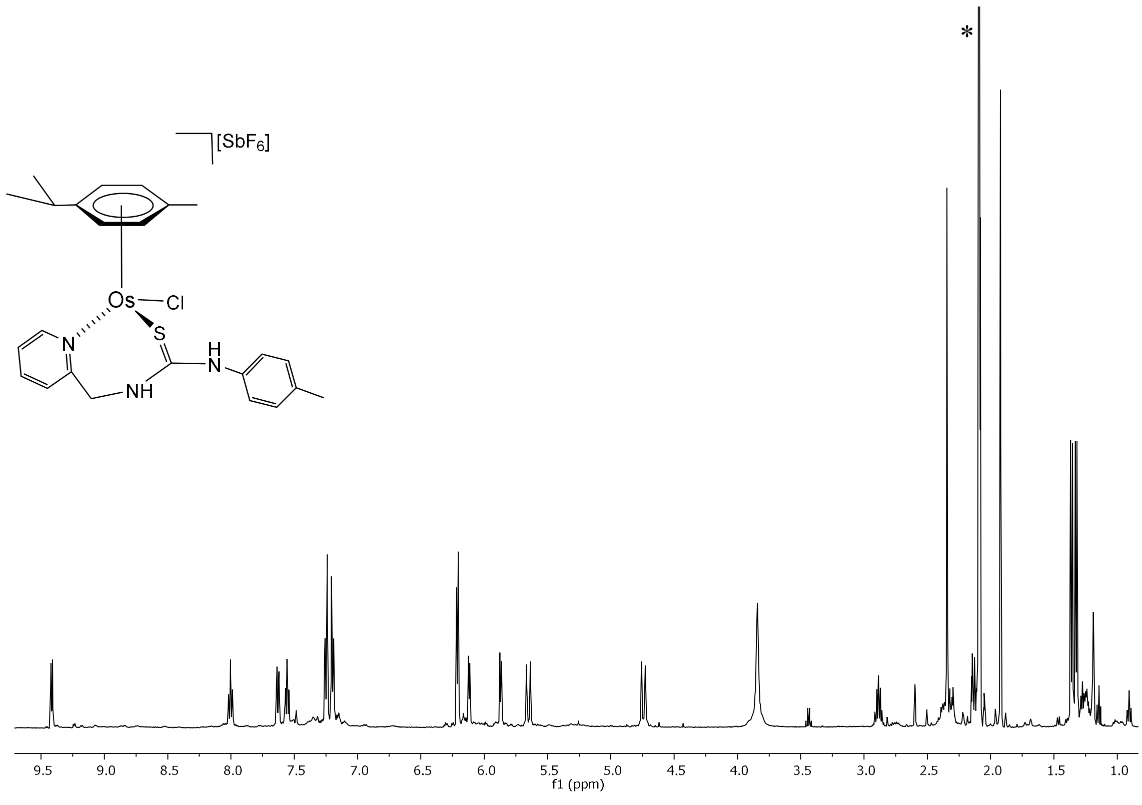

Figure S6. ^{1}H NMR of [(Cym)OsCl(κ^{2}N_{py},S-H_2NNS)][SbF_6] (2) (500.10 MHz, $(CD_3)_2CO$, -80 ºC)

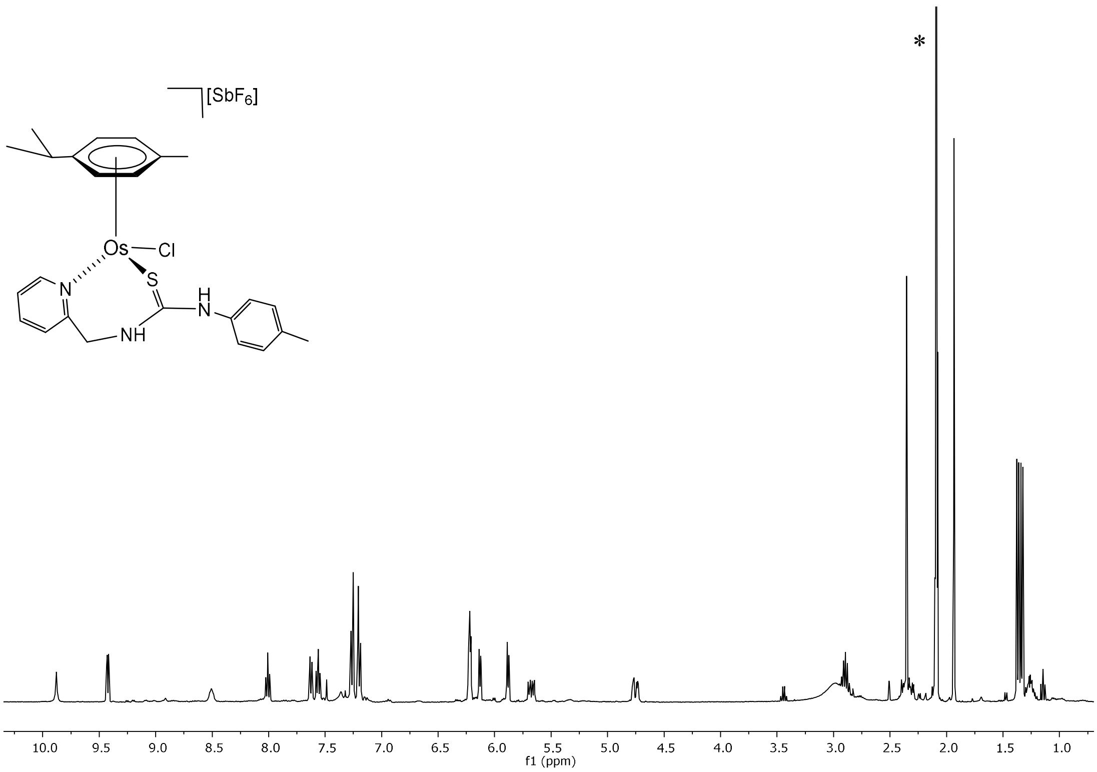

Figure S7. $^{13}C\{^{1}H\}$ **NMR of [(Cym)OsCl(κ^{2}N_{py},S-H_2NNS)][SbF_6] (2) (125.77 MHz, $(CD_3)_2CO$, RT)**

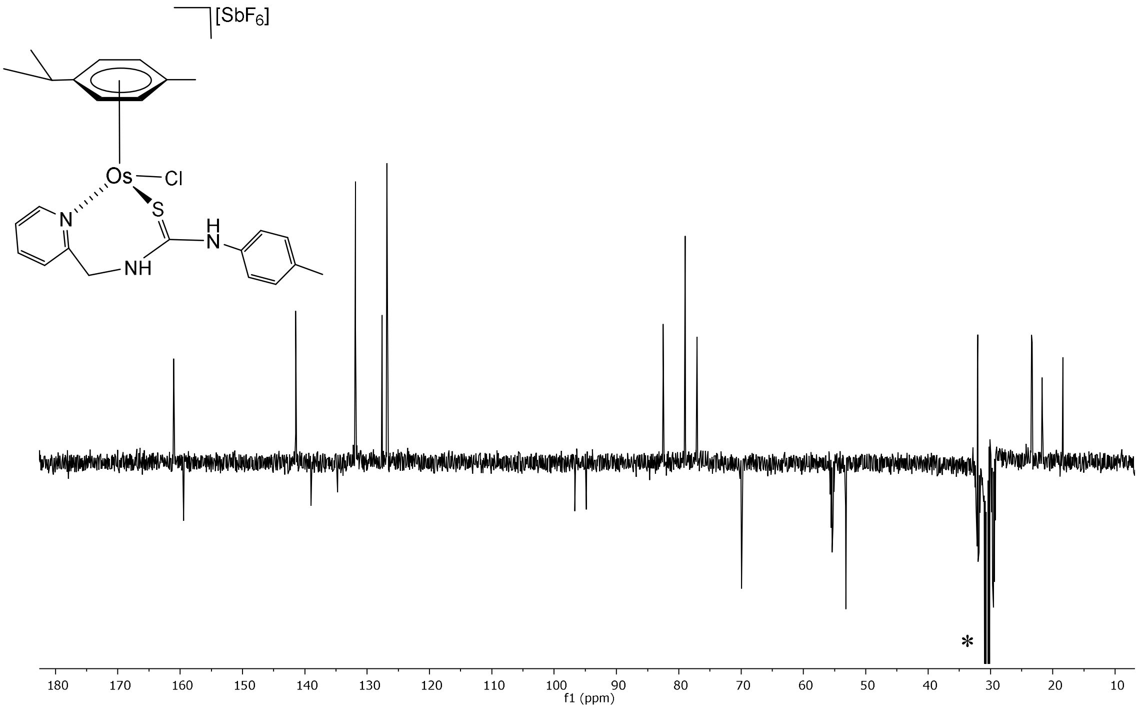

Figure S8. ^{1}H **NMR of 3a-3e (500.10 MHz, $(CD_3)_2CO$, RT)**

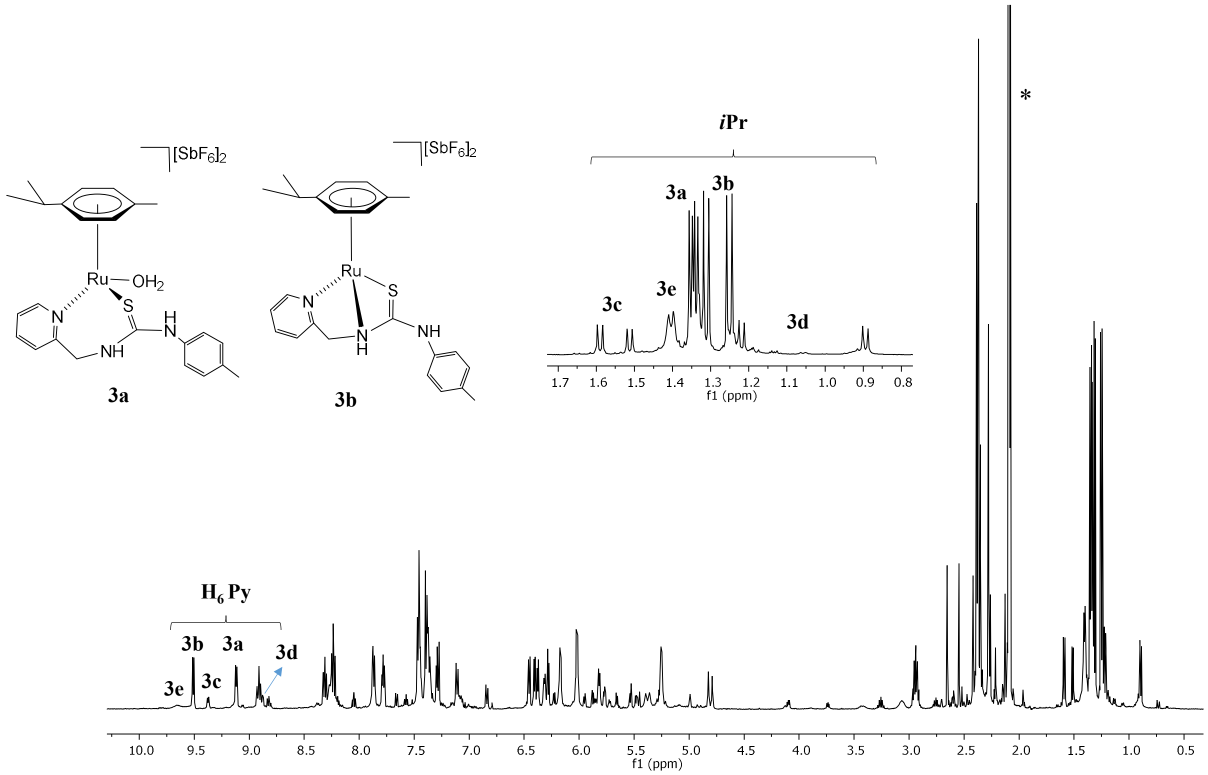

Figure S9. $^{13}C\{^{1}H\}$ **NMR of 3a-3e (125.77 MHz, $(CD_3)_2CO$, RT)**

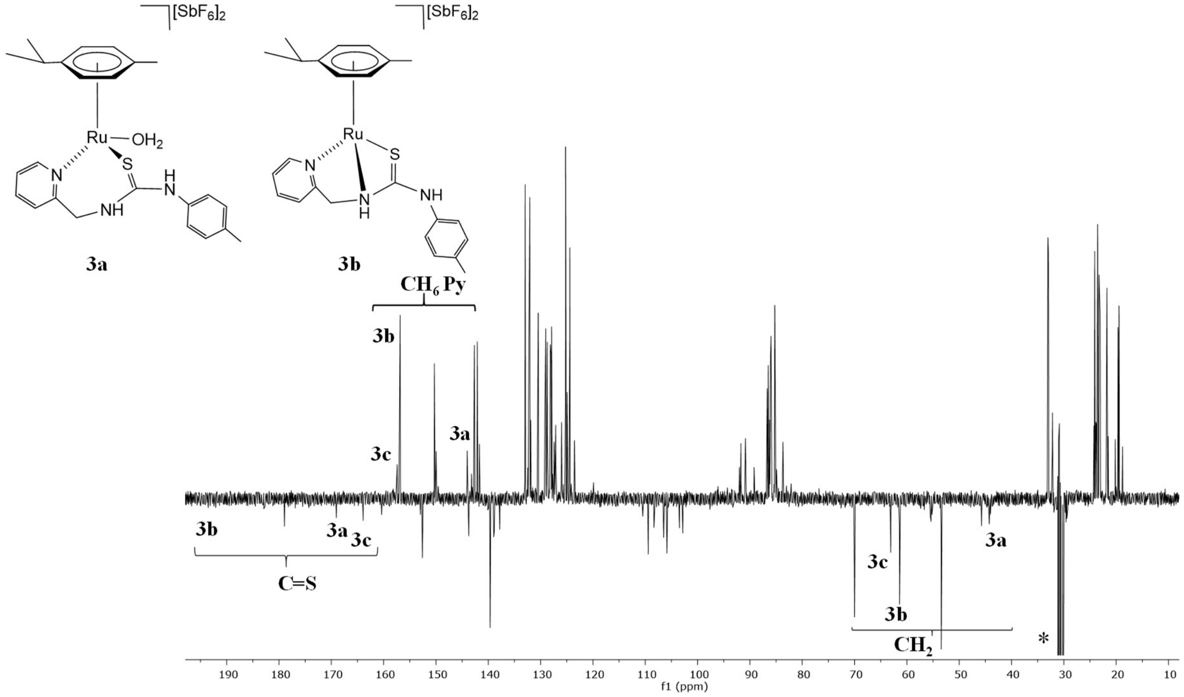

Figure S10. ^{1}H **NMR of [(Cym)Ru(NCMe)(κ^2*N*$_{py}$,*S*-H_2NNS)][SbF_6]$_2$ (3f) (400.16 MHz, CD_2Cl_2, RT)**

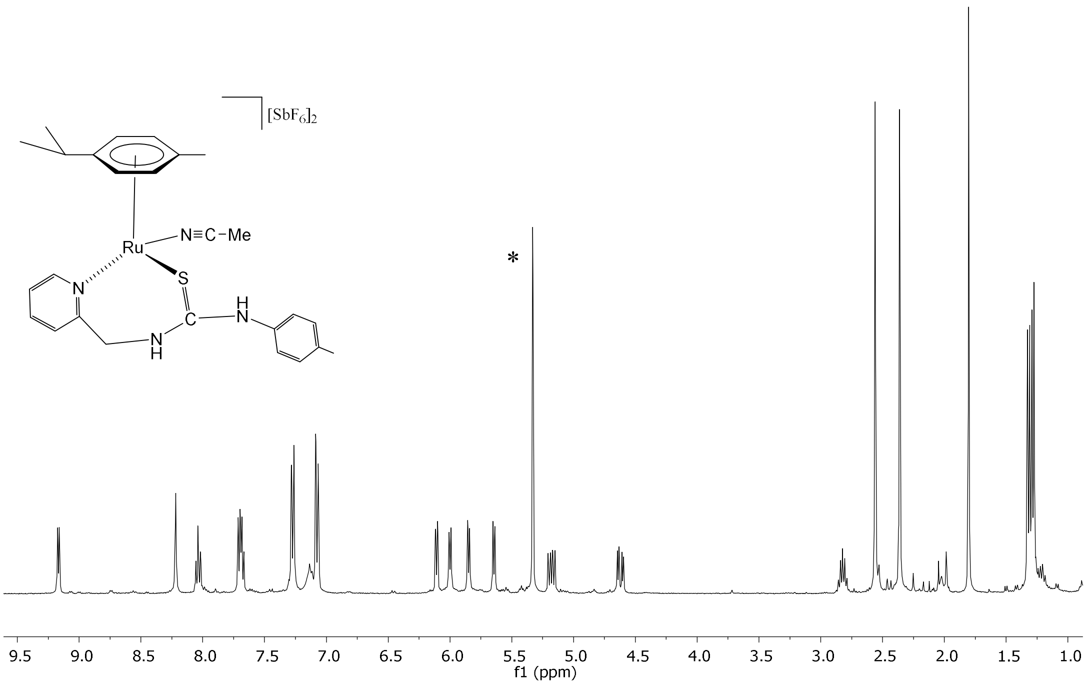

Figure S11. $^{13}C\{^{1}H\}$ **NMR of [(Cym)Ru(NCMe)(κ²*N*py,*S*-H₂NNS)][SbF₆]₂ (3f) (125.77 MHz, CD₂Cl₂, RT)**

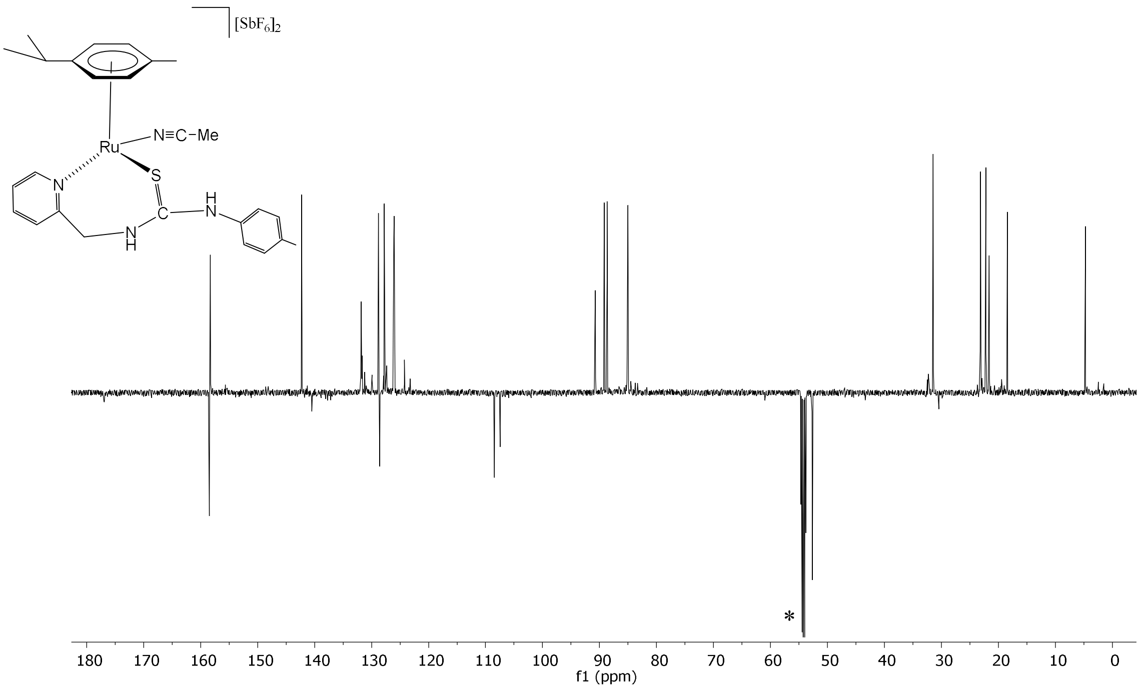

Figure S12. ^{1}H **NMR of [(Cym)Os(NCMe)(κ²*N*py,*S*-H₂NNS)][SbF₆]₂ (4a) (500.10 MHz, CD₂Cl₂, RT)**

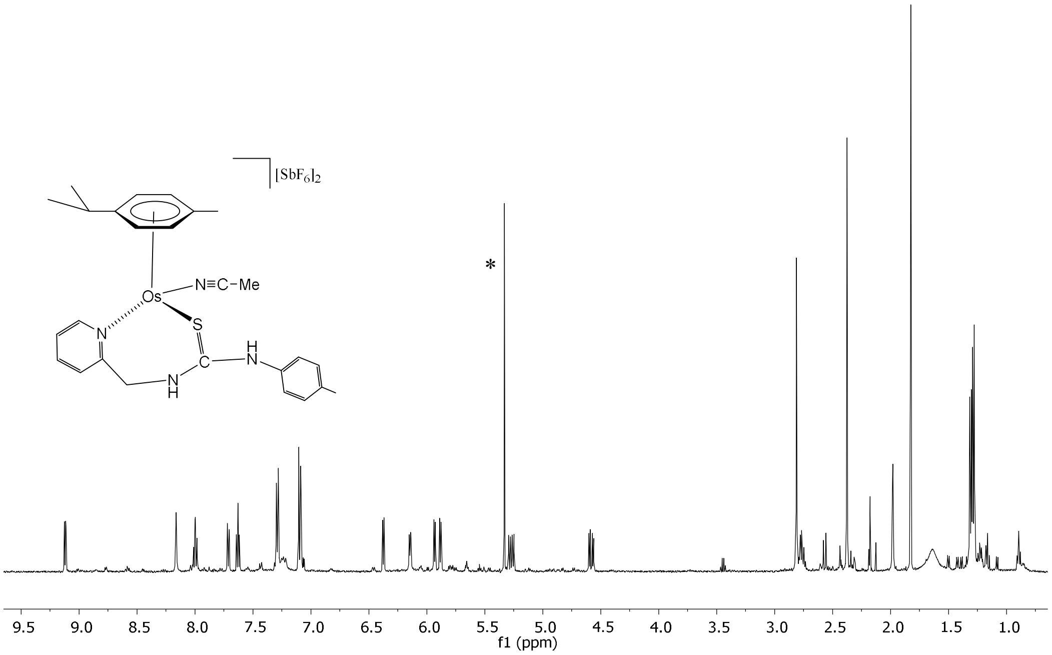

Figure S13. $^{13}C\{^{1}H\}$ **NMR of [(Cym)Os(NCMe)(κ²*N*py,*S*-H₂NNS)][SbF₆]₂ (4a) (125.77 MHz, CD₂Cl₂, RT)**

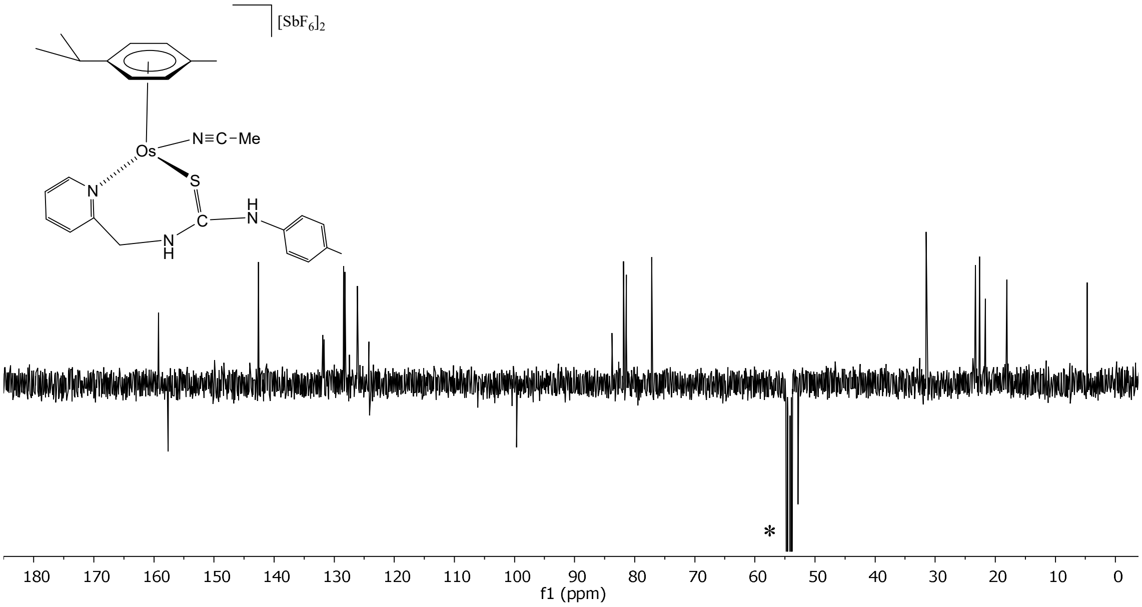

Figure S14. ^{1}H **NMR of 5a-5c (500.10 MHz, THF-*d*$_8$, RT)**

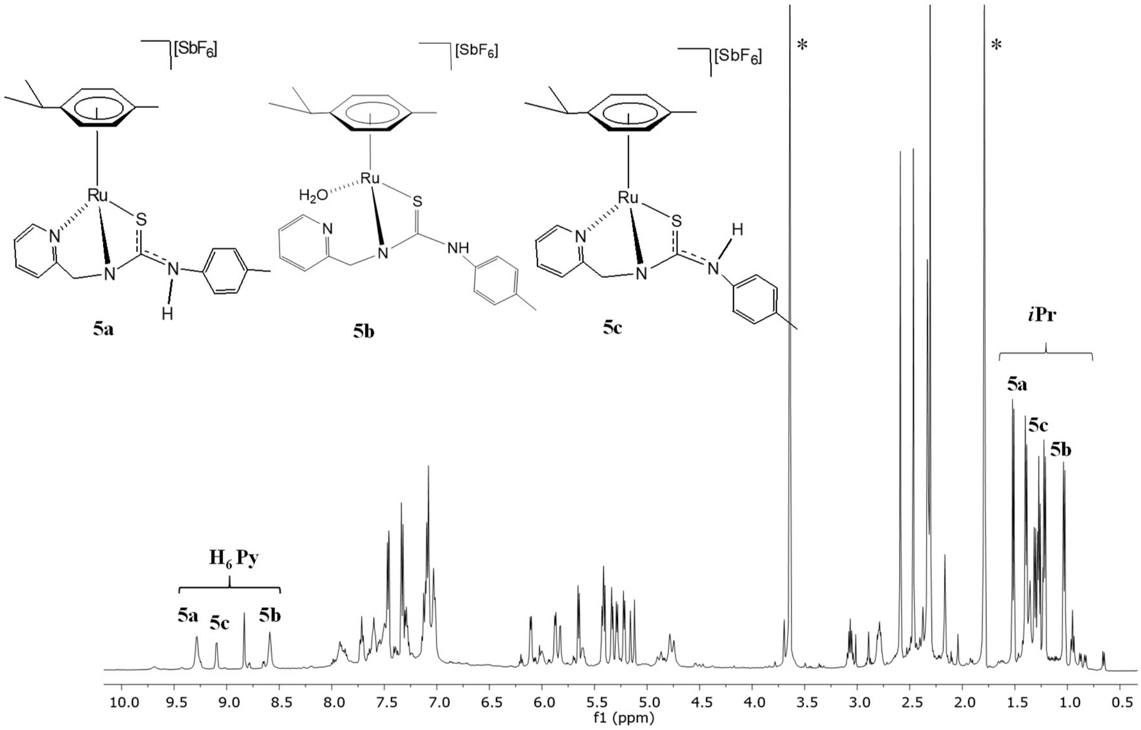

Figure S15. $^{13}C\{^{1}H\}$ **NMR of 5a-5c (125.77 MHz, THF-*d*$_8$, RT)**

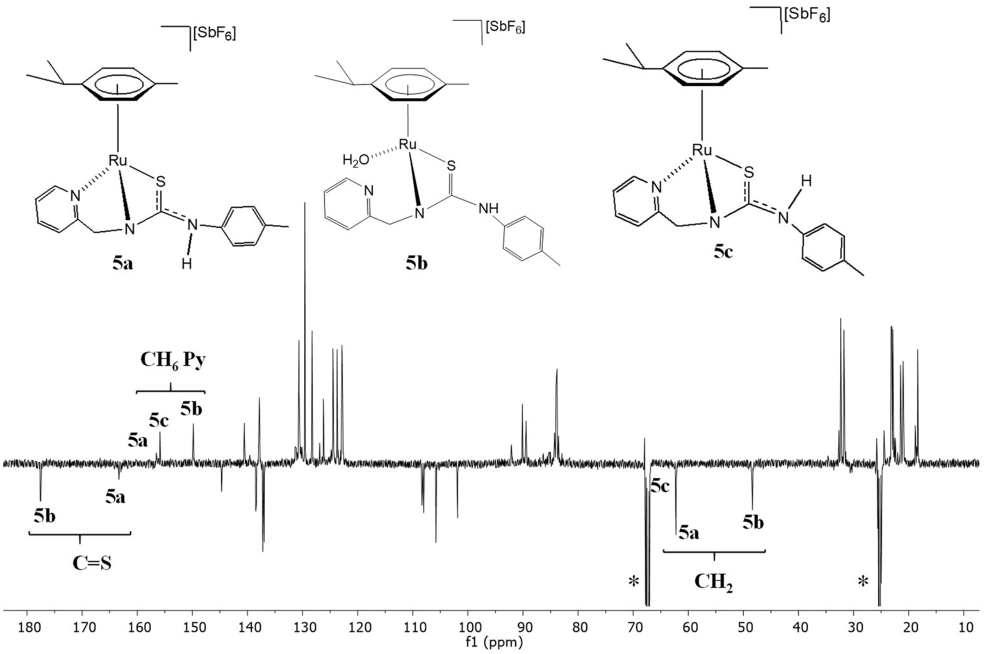

Figure S16. ^{1}H **NMR of [(Cym)Ru(κ^3*N*$_{py}$,*N*$_{amide}$,*S*-HNNS)][SbF$_6$] (5c) (500.10 MHz, THF-*d*$_8$/D$_2$O, 0.35/0.10, RT)**

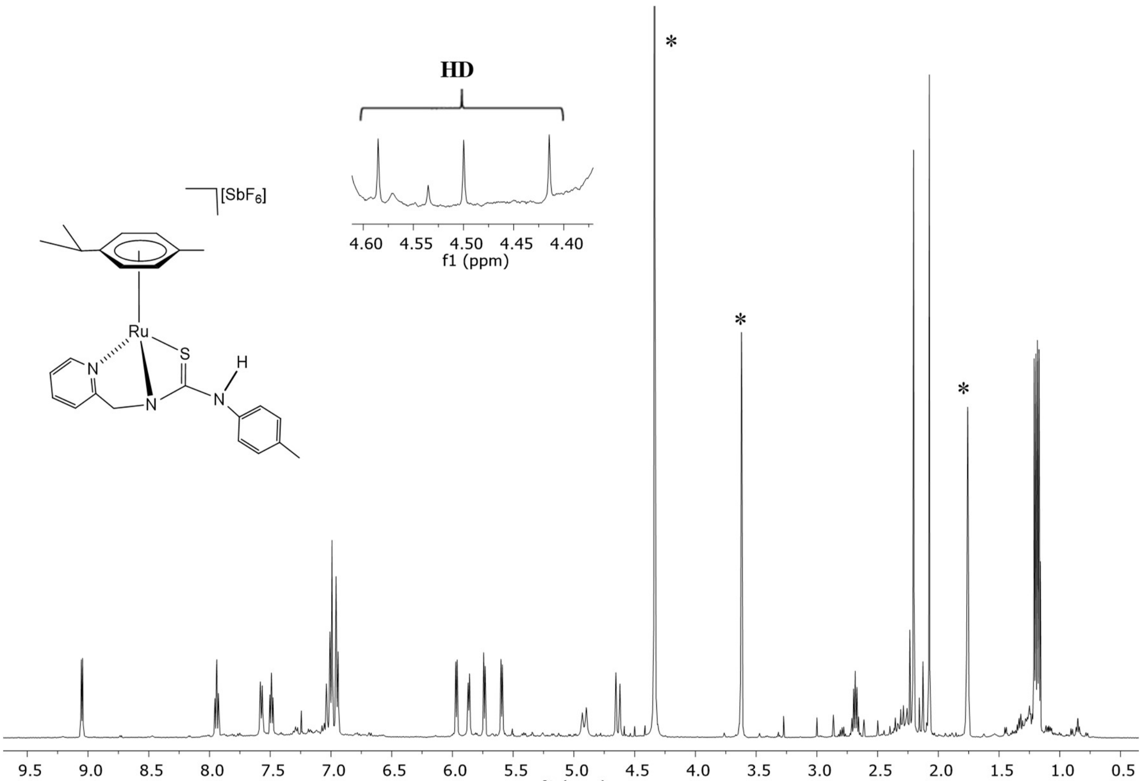

Figure S17. $^{13}C\{^{1}H\}$ **NMR of [(Cym)Ru(κ$^{3}N_{py}$,N_{amide},*S*-HNNS)][SbF$_6$] (5c) (125.77 MHz, THF-d_8/D_2O, 0.35/0.10, RT)**

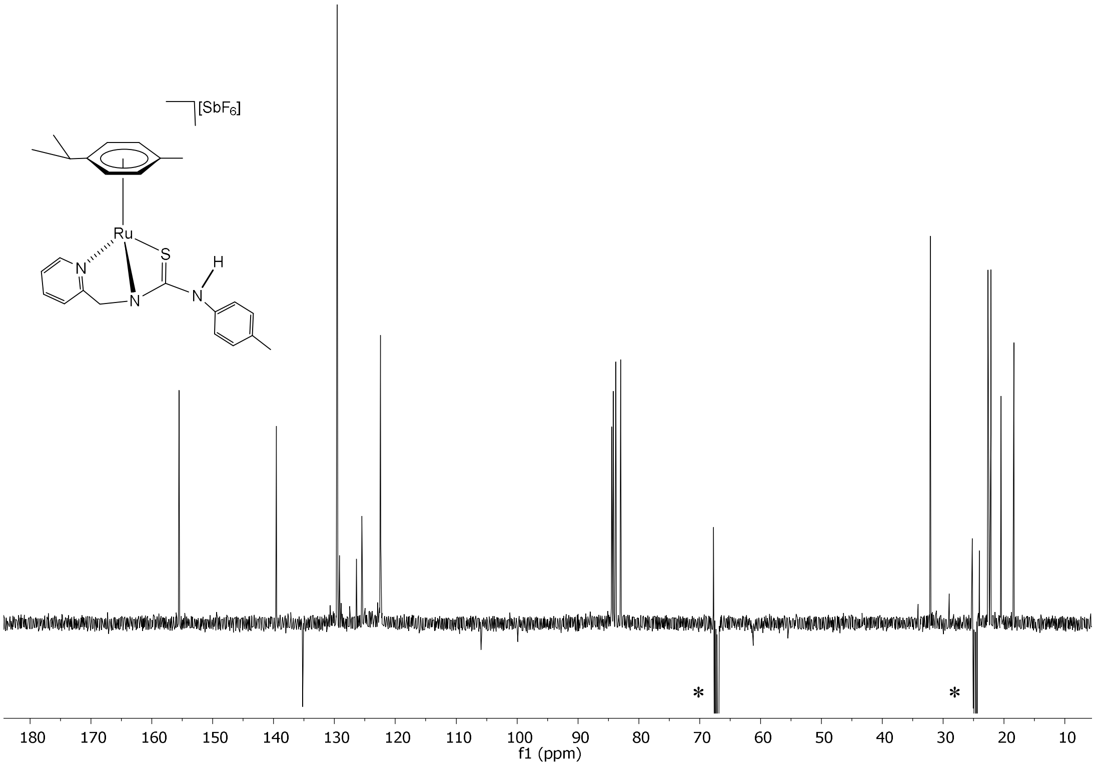

Figure S18. ^{1}H **NMR of 6a, 4b and 6c (500.10 MHz, CD_3OD, RT)**

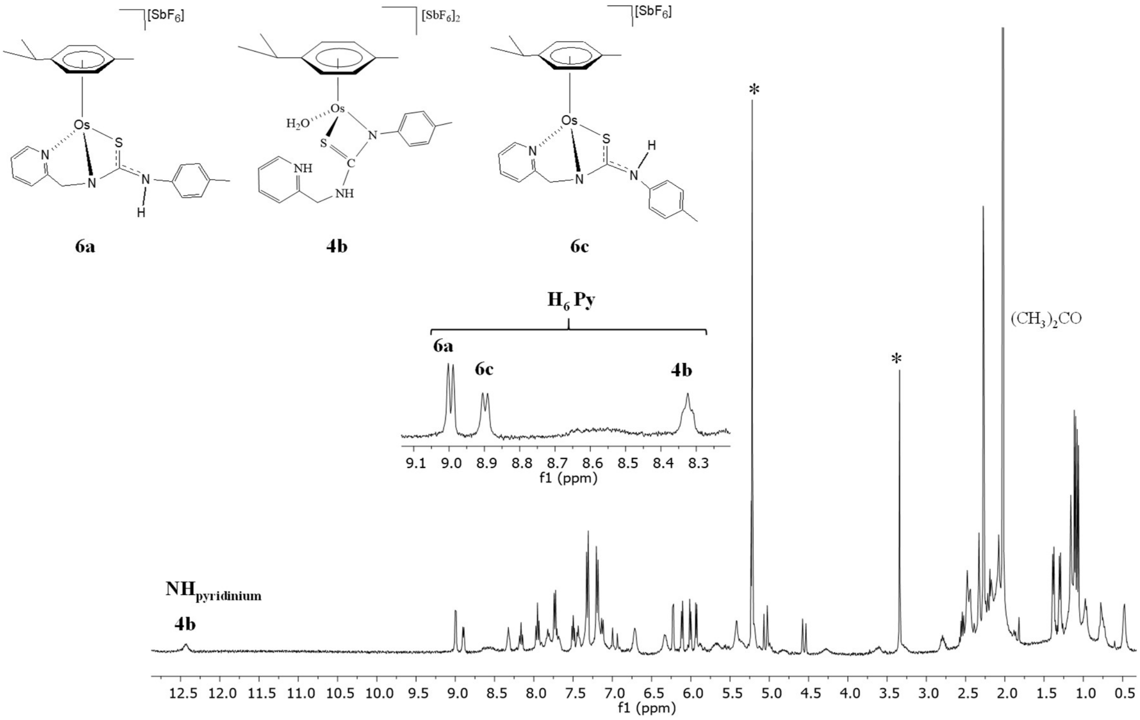

Figure S19. ^{1}H **NMR of 6a and 6b (500.10 MHz,** CD_2Cl_2**, RT)**

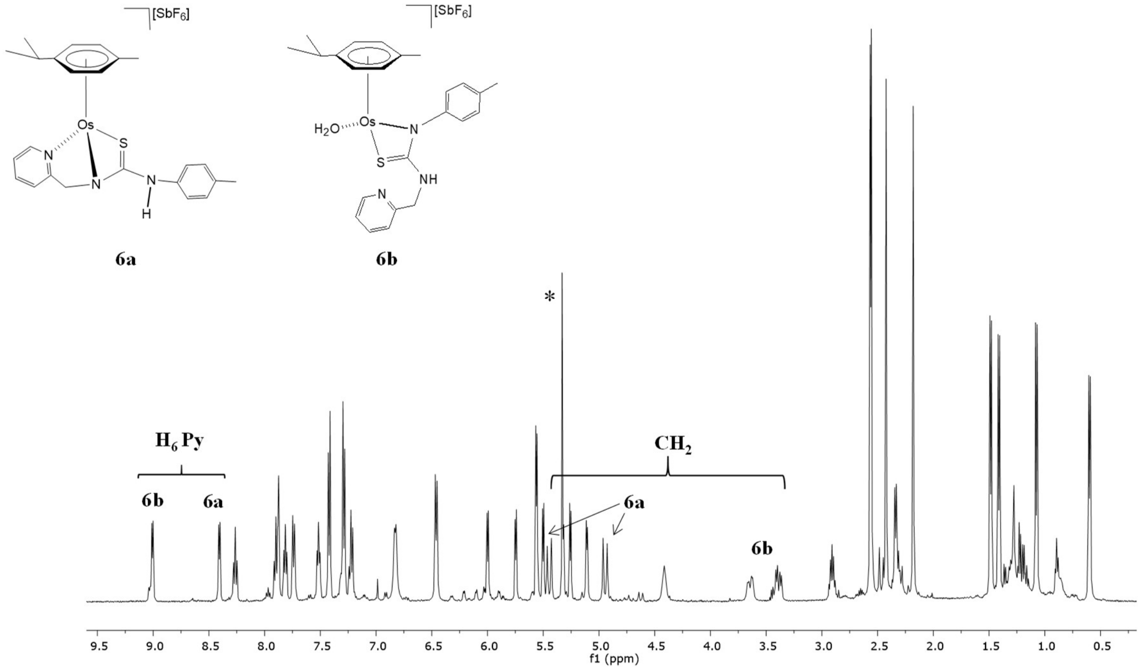

Figure S20. $^{13}C\{^{1}H\}$ **NMR of 6a and 6b (125.77 MHz,** CD_2Cl_2**, RT)**

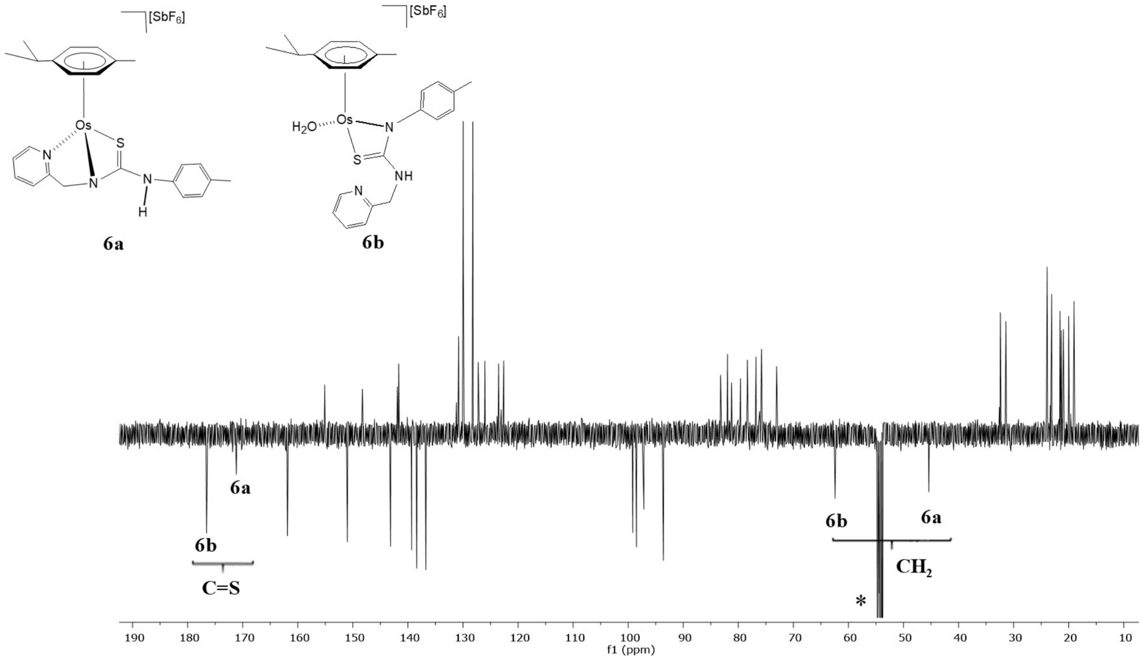

Figure S21. ^{1}H NMR of [(Cym)Os(κ^{3}N_{py},N_{amide},S-HNNS)][SbF$_6$] (6c) (500.10 MHz, CD$_2$Cl$_2$, RT)

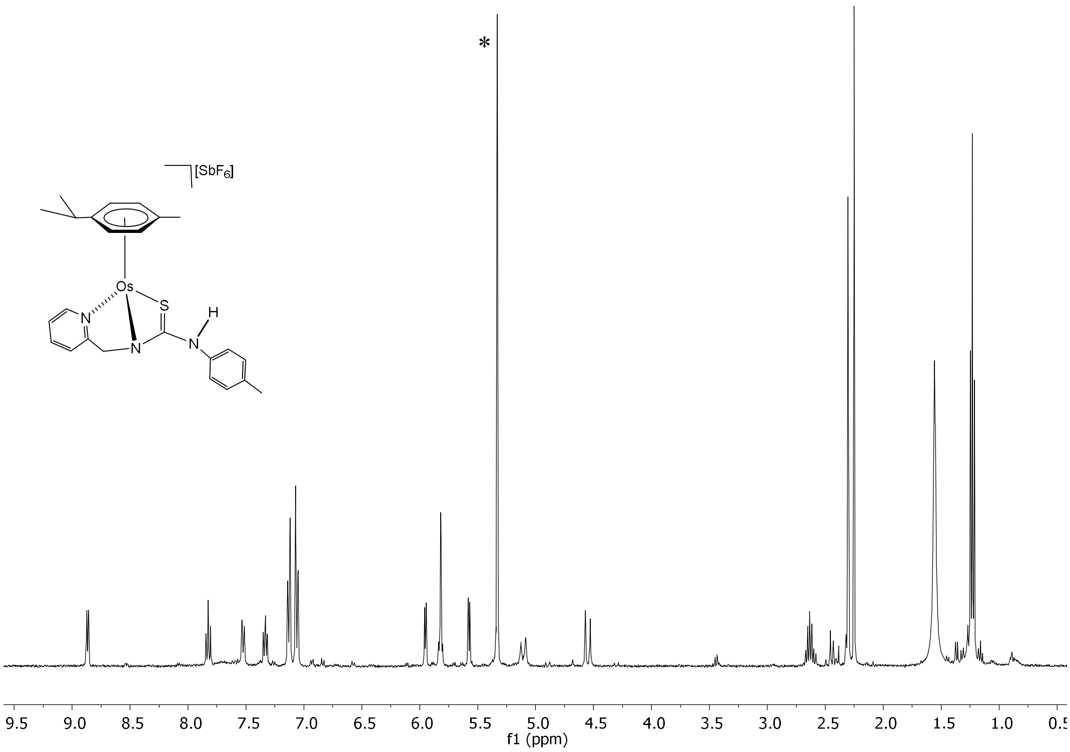

Figure S22. ^{13}C{^{1}H} NMR of [(Cym)Os(κ^{3}N_{py},N_{amide},S-HNNS)][SbF$_6$] (6c) (125.77 MHz, CD$_2$Cl$_2$, RT)

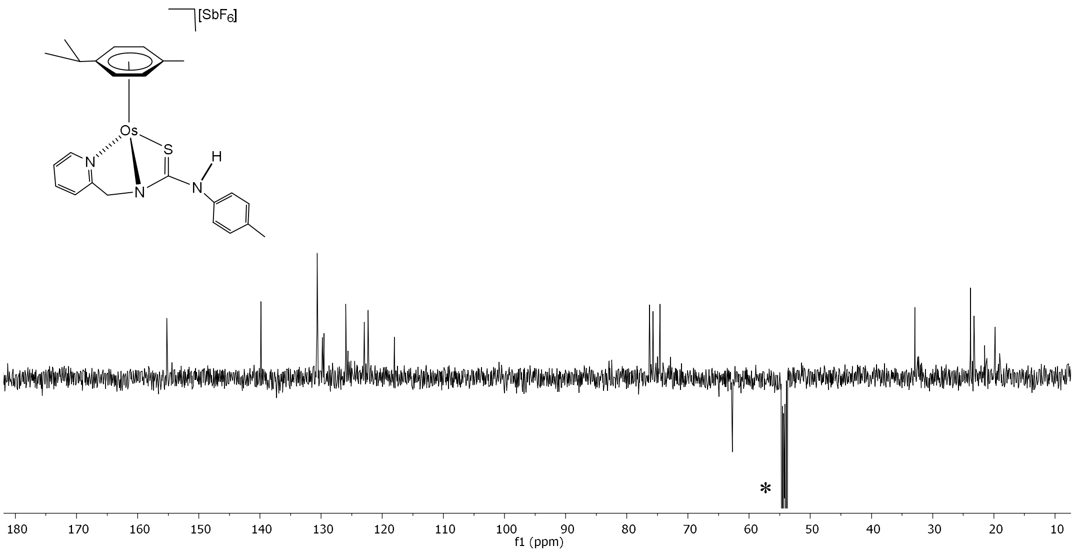
[SbF6]
Os
S
N
N
N
H
*
180
170
160
150
140
130
120
110
100
90
80
70
60
50
40
30
20
10
f1 (ppm)
